# Reactive astrocytes targeting with oral vitamin A: Efficient neuronal regeneration for Parkinson's disease treatment and reversal of associated liver fibrosis

**DOI:** 10.1111/cns.14179

**Published:** 2023-03-22

**Authors:** Nesrine Saeid El‐Mezayen, Mennat‐Allah Magdy Attia, Mohanad Yehia Shafik, Hadeer Galal Gowied, Hadeer Ahmed Abdel‐Aal, Sara Medhat Abdel‐Hady, Mostafa Said Ghazy

**Affiliations:** ^1^ Department of Pharmacology, Faculty of Pharmacy Pharos University in Alexandria Alexandria Egypt; ^2^ Faculty of Pharmacy Pharos University in Alexandria Alexandria Egypt

**Keywords:** astrocytes, liver fibrosis, Parkinson's disease, reprogramming, vitamin A

## Abstract

**Introduction:**

A recent approach to cure neurodegenerative diseases is to reprogram fibroblasts into functioning neurons using multiple exogenous transcription factors (TFs) and micro‐RNAs. Administering agents that can endogenously induce these TFs may bypass the limitations of this approach. Astrocytes may represent a part of the extrahepatic‐stellate system involved in vitamin‐A (V_A_) homeostasis. Activated‐stellate cells lose their V_A_‐storage capacity, and this was previously applied for hepatic‐stellate cells (HSCs) targeting to treat liver fibrosis. Accordingly, it is hypothesized that Parkinson's disease (PD) may be coupled with retinoid depletion that may extract V_A_ from V_A_‐rich‐HSCs triggering liver fibrosis. Thus, V_A_ administration may selectively target V_A_‐deficient reactive astrocytes and HSCs. Besides, V_A_ has the regenerative capability and may induce endogenous‐TFs generation.

**Methods:**

Fluorescently labeled V_A_‐coupled liposomes (FLV) were traced using confocal laser microscope in rats with induced PD for detecting brain accumulation and uptake into fluorescently labeled astrocytes. Liver fibrosis associated with PD was assessed biochemically and histopathologically, while V_A_ deficiency was confirmed by assessing retinol‐binding protein gene expression in the brain and liver. Multiple V_A_ doses were tested for reversing PD‐associated liver fibrosis, generating TFs (involved in reprograming astrocytes/fibroblasts into different neuronal types) and capability of dopaminergic‐neurons regeneration.

**Results:**

Fluorescently labeled V_A_‐coupled liposomes revealed selective brain accumulation and uptake into astrocytes. PD was associated with significant liver fibrosis and V_A_ deficiency in the brain and liver. Furthermore, V_A_‐medium dose (VAMD) was the optimum one for reversing PD‐associated liver fibrosis, generating multiple astrocytes/fibroblasts reprogramming TFs, regenerating dopaminergic neurons, and improving PD.

**Conclusion:**

V_A_‐medium dose pursued brain targeting in PD with the potential capability of regenerating neurons and restoring dopaminergic transmission. This may place this therapy as an essential treatment in PD management protocol.

## Graphical abstract

In Parkinson's disease (PD), astrocytes lose their vitamin‐A (V_A_) content leading to V_A_ extraction from the liver and triggering liver fibrosis. V_A_ intake will be directed toward V_A_‐deficient astrocytes and restore V_A_ content in both the brain and liver. In the brain, it has a great capacity in regenerating neurons, whereas, in the liver, it would reverse liver fibrosis.

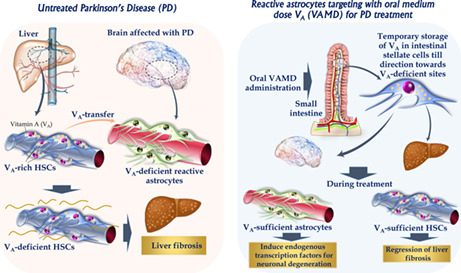

## 1 INTRODUCTION

Parkinson's disease (PD) is a common progressive neurodegenerative disorder with key features including bradykinesia, rigidity, resting tremor, and postural instability. The major neuropathologic features of PD are the loss of dopaminergic neurons in the substantia nigra pars compacta with subsequent nigrostriatal dopaminergic system dysfunction and the presence of Lewy bodies. Lewy bodies are fibrillar aggregates made up mainly of α‐synuclein that become misfolded and accumulated resulting in widely extended pathology to nervous system structures both within and beyond the CNS. Therefore, PD is associated with an array of additional motor and non‐motor features.^1^, ^2^ PD pathology remains a matter of debate and various theories have been set forth to explain PD propagation including the involvement of altered cholinergic, serotonergic, and adrenergic neurotransmissions. However, nigrostriatal dopaminergic degeneration is widely agreed to be the core PD pathological feature,^1^, ^2^ represented in the existence of a hypodopaminergic status in the midbrain with a matching loss in the dopamine transporter (DAT) and rise in the dopamine 1 (Dl) and D2 receptor densities. This may justify the initial good clinical response to levodopa.^3^, ^4^

Though progressive treatment options have been tried, they all attempt to manage PD symptoms rather than modify or cure the disease. Besides, common treatments, such as levodopa, lose effectiveness over time despite initial effectiveness Furthermore, levodopa may treat motor problems caused by low dopamine (DA) levels but not caused by other pathways involved in PD pathogenesis.^2^

A promising alternative to the standard of care for treating PD is to regenerate efficient neurons. Cumulative evidence has shown that brain fibroblasts have the ability to be reprogrammed directly to neurons. This can be accomplished by means of either getting induced pluripotent stem cells (iPSCs) or neural stem cells (NSCs) by reprogramming fibroblasts and then, obtaining neurons differentiated from iPSCs or NSCs to treat neurodegenerative disorders. These approaches have the disadvantages of the incomplete differentiation of omnipotent cells, the tendency to form tumors after transplantation, immunological rejection in addition to ethical controversy.^5^, ^6^, ^7^ Moreover, iPSCs or NSCs mostly differentiate into glial cells (astrocytes, oligodendrocytes, and microglia) rather than into a specific neuronal type.^8^ The approach that escapes these limitations is to directly convert fibroblasts into neurons depending on small molecules or defined transcription factors (TFs) such as ASCL1, LMX1, BRN2, NURR1, and FoxA2. Micro‐RNAs are another way to reprogram fibroblasts/astrocytes into neurons; they target protein‐coding mRNA transcripts via RNA silencing. MiR‐124 is commonly used in reprogramming and its expression is restricted to differentiating and mature neurons.^7^ In many studies, these TFs and MiR‐124 were successfully capable to reprogram fibroblasts into different neuron subtypes including dopaminergic, motor, serotonergic, adrenergic, and cholinergic neurons.^7^, ^9^ Unfortunately, the administration of exogenous TFs is not completely controllable and is hampered by the intricate operation, low induction efficiency and extended time consumption, which greatly limits its clinical application value.^7^ Hence, the administration of drugs that can indirectly induce endogenous TFs that are known to reprogram fibroblasts into motor or dopaminergic neurons, instead of directly using exogenous TFs, may represent a promising approach for neuronal regeneration and a cure for PD.

Vitamin A (V_A_; or retinol) is fat‐soluble vitamin that has great potential to be used in regenerative medicine; it can promote the derivation of induced pluripotent stem cells,^10^ moreover, it acts as an important survival factor for fibroblasts.^11^ Furthermore, retinoic acid (RA), the active form of V_A_, is an important modulator for neural‐related genes.^12^ Nevertheless, the ability of V_A_ to cause neuronal regeneration in PD has never been tested before and the mechanistic insights of how it may induce this regeneration are unknown.

Under normal healthy conditions, V_A_ is predominately stored in hepatic stellate cells (HSCs) which store up to 80% of total body retinol content.^13^ As a response to tissue injury, these stellate cells become activated myofibroblasts and lose their V_A_ storage capacity. It was previously proved that there is a diffuse stellate cell system and that stellate cells exist in extrahepatic sites including the kidney, colon, pancreas, lung, and heart.^14^ Within the brain, astrocytes share many structural and functional similarities to HSCs. In addition, astrocytes become activated for tissue repair in response to brain injury in many pathological conditions including PD, just like HSCs.^15^, ^16^ In addition, astrocytes have been proposed to be the source of RA for neurogenesis regulation in the adult brain.^17^, ^18^ Yet it has not been proven earlier that brain injury during PD is associated with V_A_ deficiency.

Taken together, the present study aimed to prove whether PD induction is coupled with the depletion of brain retinoids and that orally administered V_A_ would accumulate within the brain. This was done by quantifying the fluorescence intensity of fluorescently labeled V_A_‐coupled liposomes in different organs following oral administration using a confocal laser microscope in rats with haloperidol‐induced PD. In addition, retinol‐binding protein (RBP) gene expression within brain tissue was assessed in diseased and healthy conditions. The tendency of different V_A_ doses to induce endogenous TFs (ASCL1, LMX1, BRN2, NURR1 FoxA2, and MiR‐124) capable to reprogram fibroblasts into neurons was tested as a possible mechanism to induce brain regeneration. The extent of V_A_ efficacy was compared to that of the standard of care (levodopa/carbidopa) and was examined histopathologically in addition to quantification of DA, D1, D2 and DAT within brain tissue. PD markers; α‐Synuclein and ApoA1, were also assessed along with the effect on motor activity using the Rota rod apparatus.

## 2 MATERIALS AND METHODS

### 2.1 Materials

N‐[1‐(2,3‐Dioleoyloxy)propyl]‐N,N,N‐trimethylammonium‐methyl sulfate (DOTAP) was a gift from LIPOID‐Co (Nattermannallee); V_A_ (all‐trans‐retinol), egg phosphatidylcholine, cholesterol and Nile Red were from Sigma‐Aldrich; Carbidopa/levodopa from Egyphar Co. Haloperidol from Marcyrl Co.; Rat α‐Synuclein, transforming growth factor‐beta (TGF‐β) and DA ELISA Kits from CUSABIO; Rat APOA1 ELISA Kit was from Kamiya biomedical company; Hydroxyproline ELISA Kit from Mybiosource; miRNeasy Mini total and miRNA extraction kit, high capacity cDNA reverse transcription kit, Rotor‐Gene SYBR® green PCR kit and miScript SYBR® green, RT‐PCR reagents kits and universal primers were from Qiagen; Trizole plus RNA purification kit was from Invitrogen—ThermoFisher; ASCL1 (G‐9) monoclonal antibody (Catalog #sc‐374550) was purchased from Santa Cruz Biotechnology, Inc; BRN2‐specific polyclonal antibody (Catalog #18998‐1‐AP) from Proteintech; DRD1 polyclonal antibody (Catalog #720276), DRD2 (extracellular) polyclonal antibody (Catalog #PA5‐77389), FOXA2 polyclonal antibody (Catalog #PA1‐31936), NURR1 polyclonal antibody (Catalog #PA1‐4519) and alfa‐smooth muscle actin (α‐SMA) were from Invitrogen, ThermoFisher; LMX1A polyclonal antibody (Catalog #NBP2‐41193) was from Novus Biologicals USA; Goat anti‐rabbit horseradish peroxidase‐conjugated secondary antibody (Catalog #BA1055) from Wuhan Boster Biological Technology Ltd Goat anti‐mouse IgG H&L (Alexa Fluor® 555) secondary antibody was from Abcam; FastGene unstained protein marker (Catalog # MWP07) was from NIPPON Genetics; RIPA lysis buffer with protease inhibitor cocktail and BCA protein assay kit were from Beyotime Institute of Biotechnology.

### 2.2 Animals

The present study was performed on 48 female Sprague–Dawley rats of a locally‐bred strain, weighing 200 ± 10 g. The rats were purchased from and housed in the animal house of Faculty of Pharmacy, Pharos University in Alexandria (PUA), Egypt. Use of animals and experimental procedures were approved by the Ethics Committee, PUA, Egypt, in fulfillment of the NIH guidelines for the care and use of laboratory animals.

### 2.3 The experimental design

#### 2.3.1 Tracing V_A_ bio‐distribution in rats with induced PD following oral administration

These experiments were performed in order to investigate the hypothesis that PD would create a status of V_A_‐deficient brain tissue that may direct orally administered V_A_ toward the brain tissue. Thus, fluorescently labeled V_A_‐coupled liposomes (FVL) were prepared and quantitatively traced in different organs following oral administration to rats with induced PD.

##### Preparation of fluorescently labeled V_A_ ‐coupled liposomes

FVLs were prepared by applying the thin‐film‐hydration method.^19^ The utilized phospholipid mixture was phosphatidylcholine, DOTAP and cholesterol at a molar ratio 4:1:1. Nile Red fluorescent dye was added to the lipid mixture at dye‐to‐total lipid ratio (1:300).^19^ Liposomes were allowed to develop overnight and then sonicated using Sonica ultrasonic cleaner, Italy, for 15 min. V_A_ coupling was performed by mixing 200 nmol of V_A_ in DMSO with the prepared FVL suspensions by vortexing at 25°C. Excess uncoupled V_A_ was separated using a centrisart 2.5 mL concentrator of 20,000 MWCO (Sartorius). The FVL suspension was then centrifuged at 15,000 rpm for 10 min at 10°C using a cooling centrifuge. Trapped liposomes were reconstituted with deionized water.^20^ The prepared liposomes were subjected to routine in vitro characterization of nanoparticles; entrapment efficiency (EE) and in vitro‐release testing in simulated gastric fluid and methanolic phosphate‐buffered saline (PBS) pH 6.8. The prepared nanoparticles showed high EE (94.14 ± 1.5%) and excellent stability in both media.

##### Quantitative bio‐distribution testing of FVL in rats with induced PD

PD was induced in six rats using haloperidol 1.5 mg/kg i.p. once daily for 35 days^21^ (the duration for induction was set based on a pilot study). These rats were orally fed with three successive doses of FVL with 2 h dose intervals, each equivalent to 1500 IU/kg of V_A_. Half an hour after the last dose, rats were euthanized, dissected, and different organs including the brain, liver, intestine, lung, kidney, and spleen were harvested. Isolated tissue specimens were cryogenically sectioned, mounted on slides, air‐dried, and then fixed in acetone. After that, sections were washed with phosphate‐buffered saline (PBS), examined under confocal microscopy (Leica DMi8) and fluorescent images for FVL were acquired at λem 695. The intensity of red fluorescence in sections obtained from different organs was quantified using ImageJ software. These steps were repeated for another six normal rats to compare V_A_ distribution in normal versus diseased conditions.

##### Proving that activated astrocytes may act as V_A_ reservoirs in PD status

Cryogenically sectioned brain tissues prepared in the previous section were blocked, then, incubated with 200 folds diluted α‐SMA primary antibody for staining activated astrocytes. Sections were then washed with PBS and incubated with diluted goat anti‐mouse IgG H&L (Alexa Fluor® 555) secondary antibody for 30 mins at room temperature in the dark. Then, sections were washed with PBS and examined at λem 550 using confocal microscopy for locating the coincidence of FVL within the activated astrocytes.

#### 2.3.2 Experimental grouping

**FIGURE 1:**
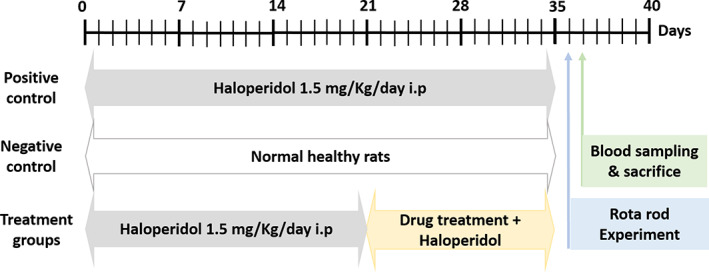
Schematic presentation of the experimental timeline.

PD was induced, as mentioned in section Quantitative bio‐distribution testing of FVL in rats with induced PD, in thirty rats that were divided into five groups (each comprising six rats) as follows: The positive control group; rats with induced PD received 1 mL/day corn oil orally. Levodopa/Carbidopa 4:1 (C/L)‐treated group; rats received 12 mg/kg orally dissolved in deionized water.^22^ Low dose V_A_ (VALD)‐treated group; rats received 1500 IU/kg/day V_A_ orally. Medium dose V_A_ (VAMD)‐treated group; rats received 3000 IU/kg/day V_A_ orally.^23^ High dose V_A_ (VAHD)‐treated group; rats received 4500 IU/kg/day V_A_ orally. Corn oil was used as a vehicle for dissolving different V_A_ doses. Administration of different therapeutic treatments and vehicles began after 21 days of induction and continued for 14 days in line with the induction. These five groups were compared to a group of six healthy normal rats. A schematic presentation of the experimental design is demonstrated in Figure 1.

#### 2.3.3 Motor activity testing at the end of the treatment period (Rota rod assay)

The experiment involved placing rats on a five‐cm diameter rod, rotating continuously at 6 rpm. Rats of different groups were trained to exercise using the apparatus prior to the actual experiment and data recording. Neurological impairment is generally indicated by loss of equilibrium (falling), assessed by measuring the latency to fall in seconds, in three consecutive tests performed over a maximum of three‐min period.

#### 2.3.4 Blood and tissue sampling at the end of the experimentation period

At the end of the experimentation period, all rats were anesthetized using desflurane anesthesia. Blood samples were collected from the retro‐orbital venous plexus for sera separation and then killed by decapitation. Brains were quickly isolated, and an appropriate part was sectioned and kept in 10% formal saline for H&E and Masson's trichrome staining for histopathological examination. The remaining brain parts were stored in −80°C until performing Western blotting, quantitative real‐time PCR (qRT‐PCR), ELISA, and biochemical testing.

#### 2.3.5 Biological tests

##### Proof that PD‐associated V_A_ brain deficiency may extract V_A_ from its hepatic stores triggering hepatic fibrosis

###### Assessment of V_A_ content in liver and brain tissues

This was done by determining gene expression of RBP in liver and brain tissues by one‐step qRT‐PCR. Total RNA was purified using Trizole plus RNA purification kit and isolated using miRNeasy Mini total and miRNA extraction kit according to manufacturer's instructions. The concentration and purity of RNA were determined spectrophotometrically at 260 and 280 nm, where the A260/A280 ratio of 1.8–2.0 corresponds to 90%–100% pure RNA. The cDNA was generated by reverse transcription using the high‐capacity cDNA reverse transcription kit. The cDNA is then amplified by thoroughly mixing reaction mixture (25 μL) comprised of 12.5 μL Rotor‐Gene SYBR Green RT‐PCR master mix, 0.25 μL Rotor‐Gene RT mix, 1 μL template RNA, and 6.5 μL RNase‐free water according to manufacturer's instructions to amplify the cDNA using sets of a specific primer for the gene (5 μL; Table 1). The data acquisition was collected during the extension step using Rotor‐Gene Q‐Pure detection, software version 2.1.0 (build 9); Qiagen. The relative expression of target genes was quantified relative to the expression of the reference gene (β‐actin) in the same sample and expressed as a normalized ratio. This was done by calculating the threshold cycles (*C*_t_) values of target genes to that of the reference using the ΔΔ*C*_t_ method.^24^

**TABLE 1:** Used oligonucleotide primers in the present study for RT‐PCR.

Primer		Sequence	Accession number
RBP1	Forward	5′‐TTCAACGGGTACTGGAAGAT‐3′	NM_012733.5
	Reverse	5′‐TCCTGCACGATCTCTTTGTC‐3′	
Mir‐124	Forward	5′‐TCTCTCTCCGTGTTCACAGC‐3′	NR_031866.1
	Reverse	miScript Universal Primer “supplied with the kit”	
DAT	Forward	5′‐TCTCGGACAGTTCAACAGAG‐3′	NM_012694.2
	Reverse	5′‐GCCCACGTAGAAAGAGATGA‐3′	
β‐Actin	Forward	5′‐ATCATTGCTCCTCCTGAGCG‐3′	NM_031144.3
	Reverse	5′‐GAAAGGGTGTAAAACGCAGCTC‐3′	
RNU6‐2		Gene global Id: MS00033740	NR_125730.1

#### Assessment of PD‐associated hepatic fibrosis

##### Histopathological evaluation of liver fibrosis

Parts of the liver were sliced, immersed in 10% formalin‐saline solution, dehydrated and then were paraffin‐embedded and 3–4 μm sections were cut, deparaffinized, hydrated using descending grades of alcohol, and then stained using Masson's trichrome stain. Stained sections were examined by an investigator blind to the experimental group under the light microscope (Inverted Microscope ECLIPSE Ti‐S; Nikon). The amount of fibrous tissue (blue stain) in each section was semi‐quantitatively analyzed using ImageJ software and scored according to the method of Ishak, with 7 stages (0–6).^19^

##### Determination of hepatic TGF‐β and hydroxyproline levels by ELISA

Within liver tissue, TGF‐β and hydroxyproline were measured by ELISA kits according to their manufacturer's manuals. Liver tissues were homogenized (20% homogenates in PBS (pH 7.4)) using a cooling centrifuge (Centurion‐scientific‐K3 series, UK) at 15,000 rpm at 5°C for 5 min. TGF‐β and hydroxyproline were tested in the supernatant obtained following centrifugation.

###### Influence of V_A_ on endogenous transcription factors capable of reprogramming brain fibroblasts

####### Western blot analysis of endogenous transcription factors

Protein extraction was done using RIPA lysis buffer with a protease inhibitor cocktail and the protein concentration was determined using a BCA protein assay kit according to the manufacturer's instructions. Equal amounts of protein lysates (40–60 μg) were loaded and separated by 8%–12% sodium dodecyl sulfate–polyacrylamide gel electrophoresis (SDS‐PAGE), and then were electro‐transferred onto nitrocellulose membranes (Millipore). Membranes were then blocked for 2 h at 25°C with 5% skimmed milk followed by incubation with the properly diluted ASCL1 (G‐9), BRN2, FOXA2, NURR1, or LMX1A primary antibodies overnight at 4°C. Membranes were then washed with Tris‐buffered saline‐Tween‐20 buffer (TBST) for three times, each 10 min, and incubated with goat anti‐rabbit horseradish peroxidase‐conjugated secondary antibody for 1 h at 25°C. Secondary antibody‐bound proteins were visualized with an enhanced electrochemiluminescence system (GeneTools software version 4.03.05.0; Synoptics Ltd.).

####### Assessing gene expression of miR‐124 by qRT‐PCR

The same procedure as earlier mentioned in the “Proof that PD‐associated VA brain deficiency may extract VA from its hepatic stores triggering hepatic fibrosis” section, but by utilizing Primer Assays (forward primers) and the miScript SYBR Green PCR Kit, which includes the reverse primer; miScript Universal Primer, and QuantiTect SYBR Green PCR Master Mix (Qiagen, Germany). RNU6‐2 was used as a reference gene.

###### Evaluation of V_A_ efficacy in generating dopaminergic neurons

####### Assessment of brain D1 and D2 levels by Western blot

Levels of D1 and D2 receptors were determined by applying the same procedure explained under section “Western blot analysis of endogenous transcription factors” using DRD1 and DRD2 polyclonal antibodies.

####### Determination of brain DA level by ELISA

DA level within the brain tissue was measured by ELISA kit according to the manufacturer's instructions. Levels were assayed in the supernatant obtained following centrifugation of the homogenized brain tissue (20% homogenates in PBS (pH 7.4)) in cooling centrifuge (Centurion‐scientific‐K3series, UK) at 15,000 rpm at 5°C for 5 min.

####### Determination of DAT gene expression level in brain tissues by qRT‐PCR

This was done by applying the same procedure as earlier mentioned in the “Proof that PD‐associated VA brain deficiency may extract VA from its hepatic stores triggering hepatic fibrosis” section.

####### Histopathological examination of degenerated brain tissues

Formalin‐fixed brain tissue samples were dehydrated using ascending grades of alcohol (70–100%), embedded in paraffin blocks, and sectioned using a microtome to prepare 3–4 μm thick sections. Sections were then brought onto glass slides, deparaffinized using two changes of xylene, hydrated using descending grades of alcohol then brought into water. After that, slides were stained with H&E stain, left to dry, mounted in Canada balsam and covered for microscopical examination using a light microscope (Inverted Microscope ECLIPSE Ti‐S; Nikon).

Quantitative morphometric analysis of the pathological changes in substantia nigra was done for further histopathological evaluation. Degeneration was indicated by calculating the percentage of damaged neurons that either included intensely stained nuclei, separation from surrounding cytoplasm due to vacuolation or being shrunken. Sections were scored using a scale of 0–4 in which 0 = no damage, 1 = up to 25% damage, 2 = 25%–50% damage, 3 = 50%–75% damage, and 4 = more than 75% damage. Quantification was done by analyzing images from eight different fields for each rat from different groups using ImageJ software. Images were examined under the same magnification by an investigator blinded to the test group.^25^, ^26^

###### Influence on PD serum markers

####### Estimation of serum Apo A1 and α‐Synuclein by ELISA

Serum levels of Apo A1, and α‐Synuclein were measured by ELISA kits according to instructions provided by their kit suppliers.

### 2.4 Statistical analysis of the data

Data were fed and analyzed using IBM SPSS software package version 20.0. (IBM Corp).^27^ The Shapiro–Wilk test was used to verify the normality of distribution. For normally distributed quantitative data comparisons among the different groups were done using analysis of variance (ANOVA; *F* test) followed by a post hoc test (Tukey) for pairwise comparison. The quantitative data were described using mean and standard deviation. Significance of the obtained results was judged at the 5% level.^28^

## 3 RESULTS

### 3.1 Tracing V_A_ bio‐distribution in rats with induced PD following oral administration

**FIGURE 2:**
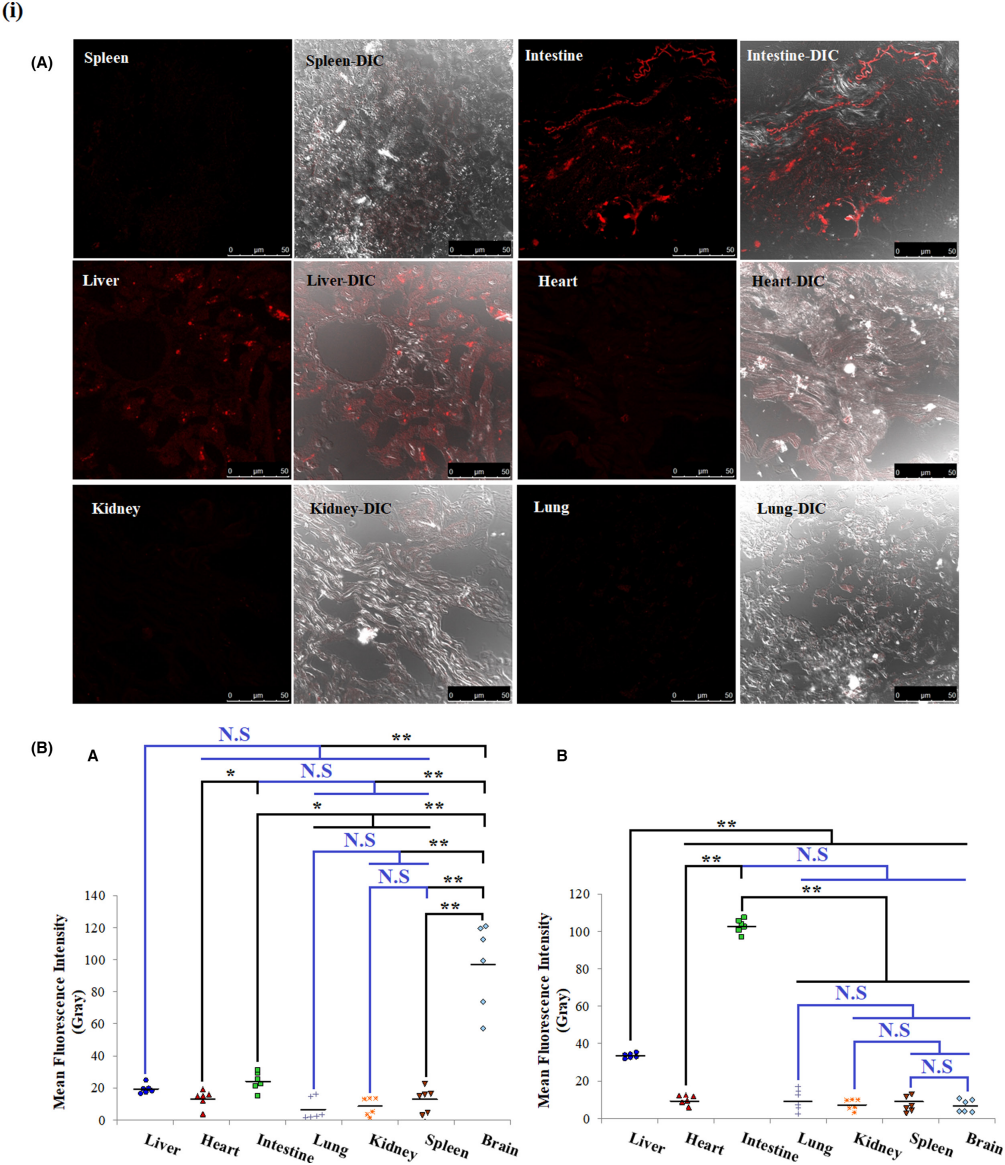
V_A_ bio‐distribution in rats with induced PD following oral administration of FVL. (A) Confocal microscope micrographs of different frozen organs sections of rats with induced PD following multiple FVL dosing; (B) Comparison between mean fluorescence intensities of different organs isolated from rats with induced PD (A) and normal (B). ANOVA test was used to compare between the different groups with post hoc test (Tukey) to compare different groups. *: Statistically significant at *p* ≤ 0.05, **: Statistically significant at *p* ≤ 0.001, N: Statistically non‐significant (*p* > 0.05), *n* = 6; all results are presented as mean ± SD. Sections were examined under confocal microscopy (Leica DMi8, Wetzlar, Germany) and fluorescent images for FVL were acquired at λem 695. The intensity of red fluorescence was quantified using ImageJ software. FVL, oral fluorescently labeled V_A_‐coupled liposomes; PD, Parkinson's disease; V_A_, vitamin A.

Tracing the bio‐distribution of V_A_ was done following multiple dosing of oral fluorescently labeled V_A_‐coupled liposomes (FVL) in rats with induced PD and freezing different organ tissues. Upon examining frozen tissue sections using confocal microscopy, considerable fluorescence was observed in brain tissue sections that far exceeded detected fluorescence in other organs sections (*p* < 0.001). The second tissue sections with detectable fluorescence in rats with induced PD were those obtained from the small intestine that showed significantly higher fluorescence intensity compared to sections of all other isolated organs (*p* ≤ 0.05). On the other hand, in normal rats, maximum fluorescence intensity was observed in intestinal sections, followed by liver sections and only minimum fluorescence was detected in all other organs sections. Mean fluorescence intensities of sections obtained from both the small intestine and liver of normal rats were significantly higher than those obtained from other organs (*p* < 0.001). Sections obtained from hearts, lungs, kidneys, and spleens of either normal rats or rats with induced PD revealed comparable fluorescence intensities with no statistically significant difference between different organs (*p* > 0.05), Figure 2.

### 3.2 Proving that activated astrocytes may act as V_A_ reservoirs in PD status

**FIGURE 3:**
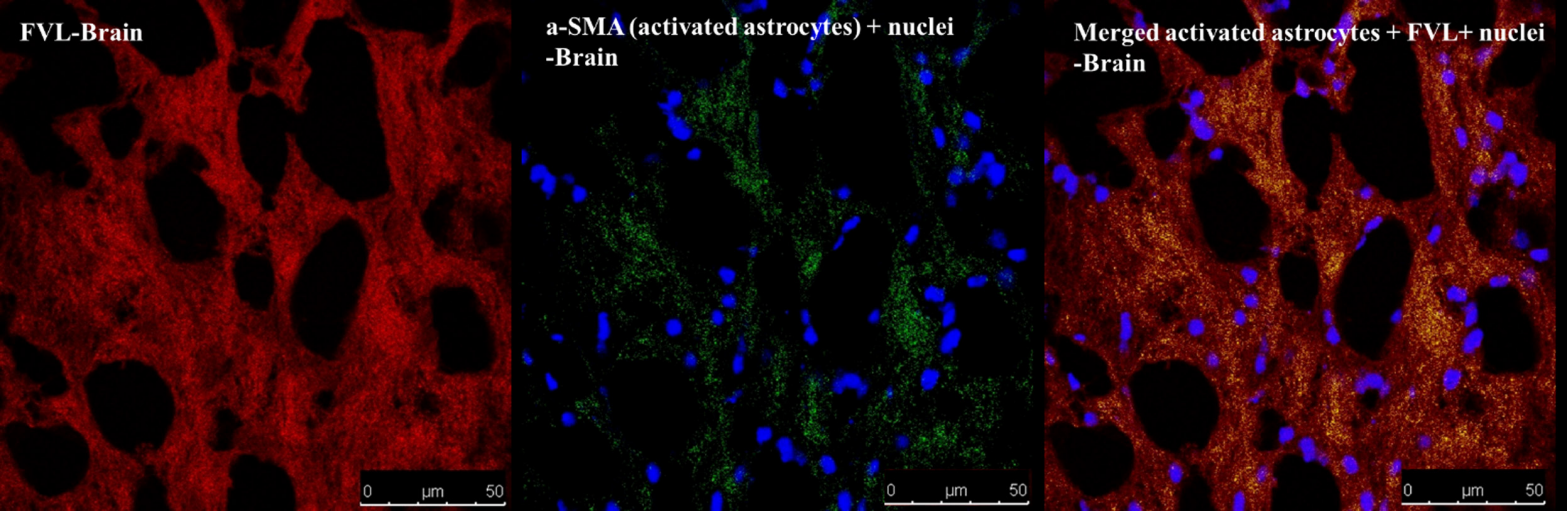
Localization of V_A_ within brain tissue of rats with induced PD following oral administration of FVL. Sections were examined under confocal microscopy (Leica DMi8) ×640 and fluorescent images for FVL, a‐SMA and nuclei were acquired at λem 695, 550 and 461, respectively. The merged images show the yellow color area by overlaying images of FVL and a‐SMA. FVL, oral fluorescently labeled V_A_‐coupled liposomes; V_A_, vitamin A.

Within brain tissue sections of rats with induced AD, activated astrocytes were immunoflourescently labeled using a‐SMA antibody (green color). Confocal laser microscopy micrographs revealed the diffuse pattern of astrocytes distribution within the brain tissue sections. Furthermore, the distribution of FVL was found to coincide with activated astrocytes in brain areas rich in activated astrocytes, Figure 3.

### 3.3 Proof that PD‐associated V_A_ brain deficiency may extract V_A_ from its hepatic stores triggering hepatic fibrosis

#### 3.3.1 Assessment of V_A_ content in liver and brain tissues

**FIGURE 4:**
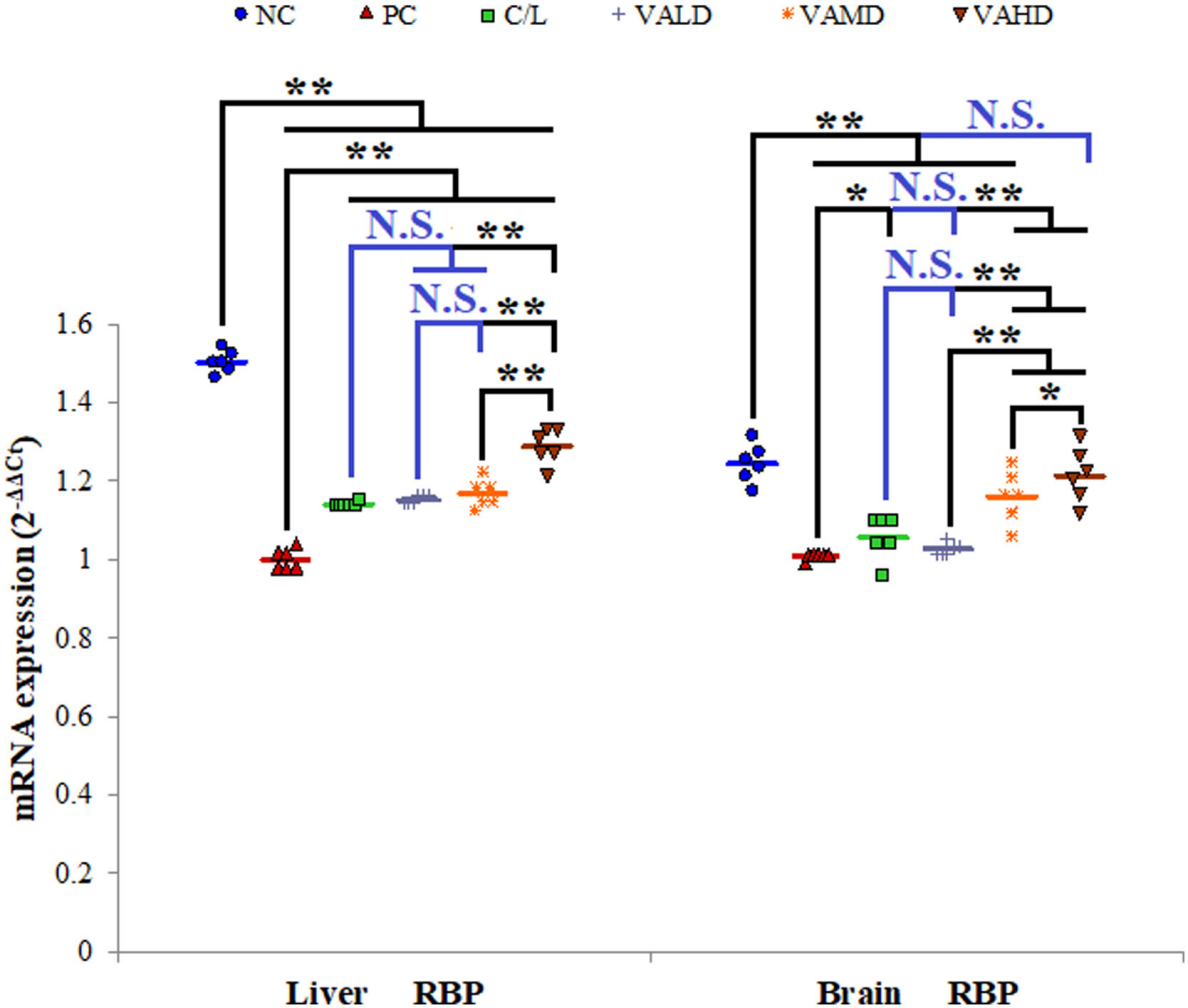
Comparison between the different studied groups according to RBP gene expression in liver and brain tissues. ANOVA test was used to compare between the different groups with post hoc Test (Tukey) and post hoc test to compare different receptors for the same group. *: Statistically significant at *p* ≤ 0.05, **: Statistically significant at *p* ≤ 0.001, NS: Statistically non‐significant (*p* > 0.05), *n* = 6; all results are presented as mean ± SD. Positive control group received haloperidol 1.5 mg/kg IP once daily for 35 days and 1 mL corn oil orally starting from Day 21. All drugs or vehicles were given as daily 1 mL oral injection for a treatment period of 14 days starting from Day 21 of induction. VALD, VAMD & VAHD were given at doses of 1500, 3000, and 4500 IU/kg/day vitamin A orally dissolved in corn oil. C/L, carbidopa/levodopa; NC, normal control; PC, positive control; RBP, retinol binding protein; VAHD, vitamin A‐high dose; VALD, vitamin A‐low dose; VAMD, vitamin A‐medium dose.

In the current investigation, in normal rats, hepatic RBP gene expression was higher in liver tissue than in brain tissue. The expression of RBP in both brain and liver tissues was significantly higher in normal rats than in rats with induced PD (*p* ≤ 0.001). Comparing RBP expression in normal rats with induced PD, it was noted that the percentage reduction in hepatic RBP expression (~33.4%) was more than in brain RBP expression (~19.7%). Supplementation with different V_A_ doses significantly increased hepatic RBP gene expression, whereas only medium and high V_A_ doses significantly increased brain RBP compared to untreated rats with PD (*p* ≤ 0.001). C/L‐ and VALD‐treated groups revealed the least gene expression of RBP in brain and liver tissues with no statistically significant difference between them (*p* > 0.05). The highest RBP gene expression in both brain and liver tissues was observed with the VAHD‐treated group with a statistically significant difference between this group and other treated groups (*p* ≤ 0.05). Of note, VAHD supplementation restored brain RBP gene expression levels with no statistically significant difference between this group and normal rats, Figure 4.

#### 3.3.2 Assessment of PD‐associated hepatic fibrosis

Assessment of liver fibrosis was done histopathologically; by examination of Masson's‐trichrome‐stained liver tissue sections and semi‐quantitatively analyzing the amount of fibrous tissue (blue color) using ImageJ software, and biochemically by assessing levels of TGF‐β and hydroxyproline in liver tissues.

**FIGURE 5:**
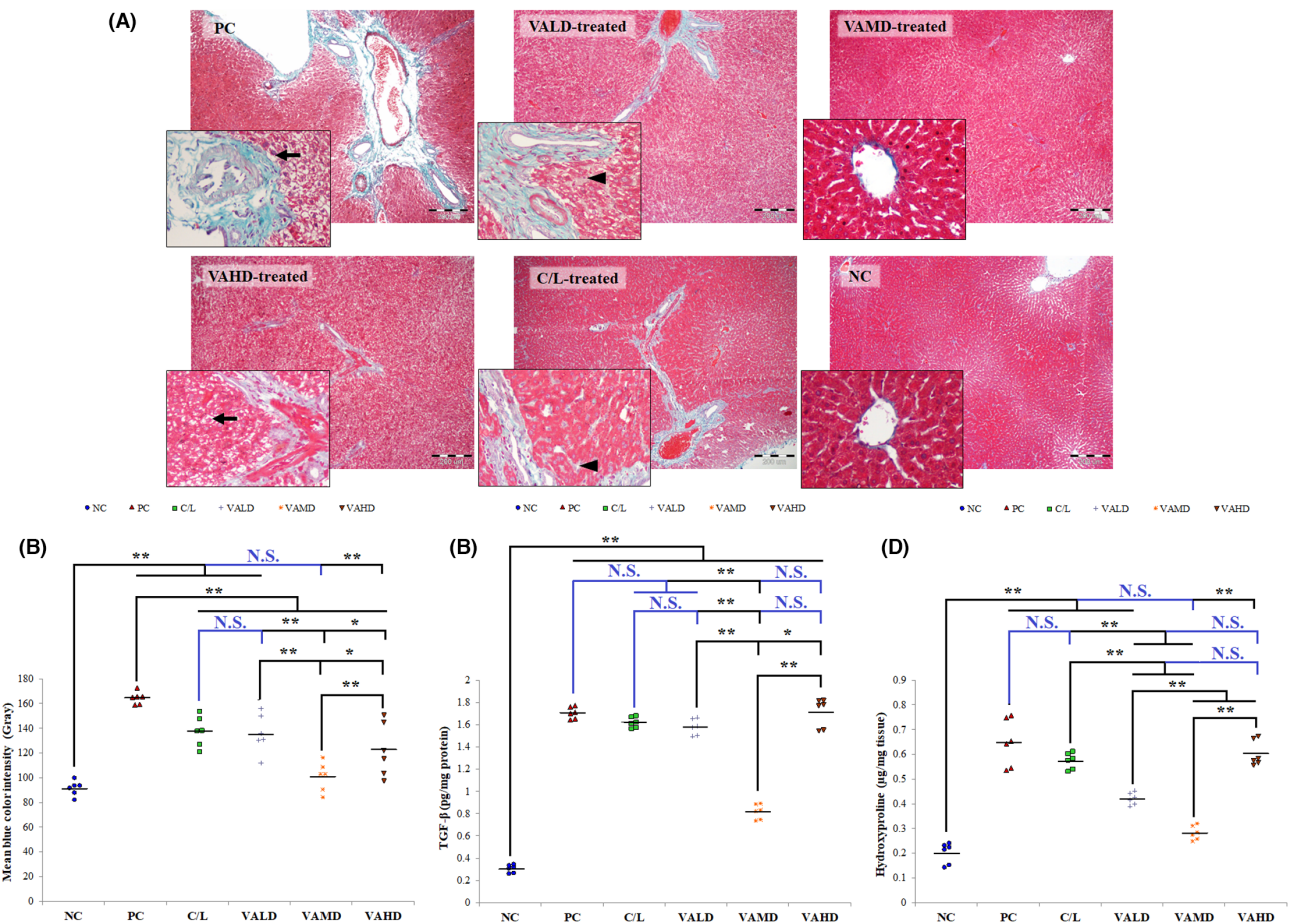
Effect of different treatments on PD‐associated hepatic fibrosis. (A) Masson's‐trichrome‐stained liver tissue sections obtained from different groups. Magnification powers of large and smaller Masson's‐trichrome‐stained liver tissue sections images are ×100 and ×400, respectively. Arrows point to necrotic and damaged hepatocytes and arrow heads point to interstitial fibrosis, (B) Semi‐quantitative analysis of the fibrous tissue (blue color) in liver sections using ImageJ software, (C) Effect of different treatment groups on hepatic TGF‐β level (pg/mg protein), (D) Effect of different treatment groups on liver hydroxyproline (μg/mg tissue). ANOVA test was used to compare between the different groups with post hoc test (Tukey) and post hoc test to compare different receptors for the same group. *: Statistically significant at *p* ≤ 0.05, **: Statistically significant at *p* ≤ 0.001, NS: Statistically non‐significant (*p* > 0.05), *n* = 6; all results are presented as mean ± SD. Positive control group received haloperidol 1.5 mg/kg IP once daily for 35 days and 1 mL corn oil orally starting from Day 21. All drugs or vehicles were given as daily 1 mL oral injection for a treatment period of 14 days starting from Day 21 of induction. VALD, VAMD & VAHD were given at doses of 1500, 3000, and 4500 IU/kg/day vitamin A orally dissolved in corn oil. C/L, carbidopa/levodopa; NC, normal control; PC, positive control; VAHD, vitamin A‐high dose; VALD, vitamin A‐low dose; VAMD, vitamin A‐medium dose.

Liver sections obtained from normal rats showed the characteristic normal architecture of liver tissue with radiating healthy hepatocytes from central veins. These sections had a very little amount of fibrous tissue at the linings of vessels (Ishak score 0), Figure 5A. Sections from untreated rats with induced PD demonstrated a significantly greater amount of fibrous deposition compared to normal rats (*p* ≤ 0.001) with hypoxic hepatocellular damage, fatty changes, and necrosis. Most of them revealed extended fibrous septa that formed bridges either porto‐portal or porto‐central, in addition, interstitial fibrous deposition was also witnessed (Ishak score 3). Liver sections of C/L‐, VALD‐ and VAHD‐treated groups revealed occasional bridging fibrosis, without multiple bridging, with prominent interstitial fibrous deposition (Ishak score 1–3). Sections obtained from VLC‐treated rats had only limited fibrous tissue around vascular areas with sporadic interstitial fibrosis (Ishak score 0–1) with no statistically significant difference in the amount of fibrous deposition between this group and NC group (*p* > 0.05), Figure 5A,B.

Mean TGF‐β and hydroxyproline concentrations were significantly higher in untreated rats with induced PD compared to normal rats (*p* ≤ 0.001). Treatment with C/L, VALD, or VAHD did not significantly reduce hepatic TGF‐β and hydroxyproline concentrations relative to the untreated group (*p* > 0.05). Only the VAMD‐treated group significantly reduced mean hepatic TGF‐β and hydroxyproline levels compared with the untreated rats with PD (*p* ≤ 0.001). The mean values of hydroxyproline following VAMD therapy did not differ significantly from those of normal values (*p* > 0.05), Figure 5C,D.

### 3.4 Influence of V_A_ on endogenous transcription factors capable of reprogramming brain fibroblasts

**FIGURE 6:**
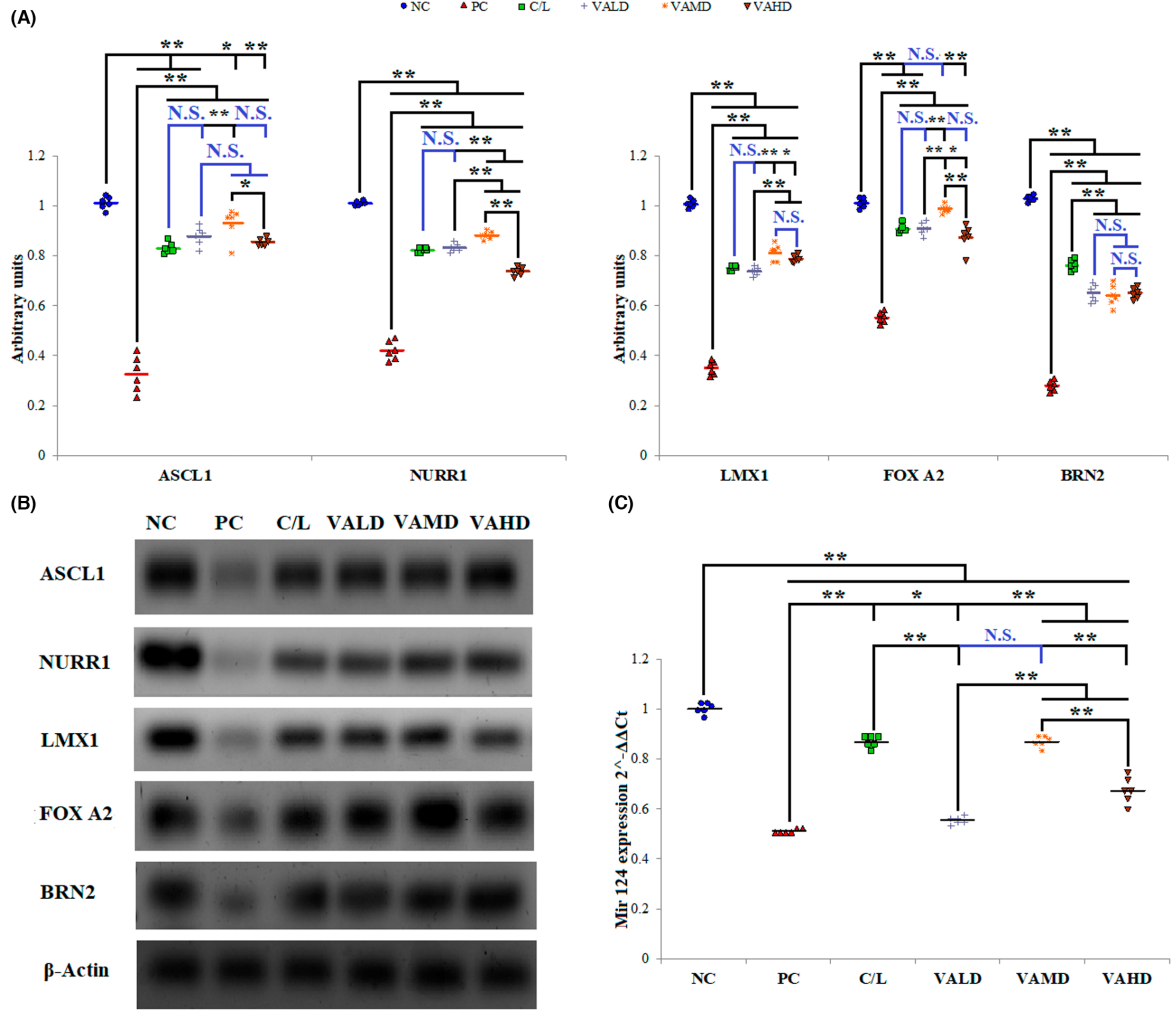
Comparison between the different studied groups according to their influence on endogenous transcription factors capable of reprogramming brain fibroblasts. (A) Comparison between different groups according to quantitative analysis of Western blot bands intensities of brain transcription factors expression, (B) Western blots for brain transcription factors expression, (C) Effect of different treatment groups on Mir‐124 gene expression assessed by qRT‐PCR. ANOVA test was used to compare between the different groups with post hoc test (Tukey) and post hoc test to compare different receptors for the same group. *: Statistically significant at *p* ≤ 0.05, **: Statistically significant at *p* ≤ 0.001, NS: Statistically non‐significant (*p* > 0.05), *n* = 6; all results are presented as mean ± SD. Positive control group received haloperidol 1.5 mg/kg IP once daily for 35 days and 1 mL corn oil orally starting from Day 21. All drugs or vehicles were given as daily 1 mL oral injection for a treatment period of 14 days starting from Day 21 of induction. VALD, VAMD, and VAHD were given at doses of 1500, 3000, and 4500 IU/kg/day vitamin A orally dissolved in corn oil. C/L, carbidopa/levodopa; NC, normal control; PC, positive control; VAHD, vitamin A‐high dose; VALD, vitamin A‐low dose; VAMD, vitamin A‐medium dose. Original blots are provided in the supplementary figures; SF1–SF8.

In the present study, induction of PD with haloperidol resulted in a significant reduction in the brain level of all assessed transcription factors compared to normal un‐diseased rats (*p* ≤ 0.001). Treatment of rats with induced PD using C/L, VALD, VAMD, or VAHD significantly increased different transcription factor expression. Different treatments affected ASCL1, NURR1, LMX1, and FOX A2 in the same manner as induction of this transcription factors expression was mostly promoted by VAMD followed by VALD and C/L and was the least in the VAHD‐treated group. Notably, FOX A2 expression in brains derived from the VAMD‐treated group was comparable to normal with no statistically significant difference observed between the two groups (*p* = 0.359). A different pattern was observed with BRN2 and Mir‐124; For BRN2, among treatment groups, C/L‐treated group revealed the greatest expression which was significantly higher than other treatment groups (*p* ≤ 0.001). For Mir‐124, both C/L and VAMD‐treated groups showed significantly greater expression than other treatment groups (*p* ≤ 0.001) and there was no statistically significant difference between both groups (*p* = 1.000), Figure 6.

### 3.5 Evaluation of V_A_ efficacy in generating dopaminergic neurons

**FIGURE 7:**
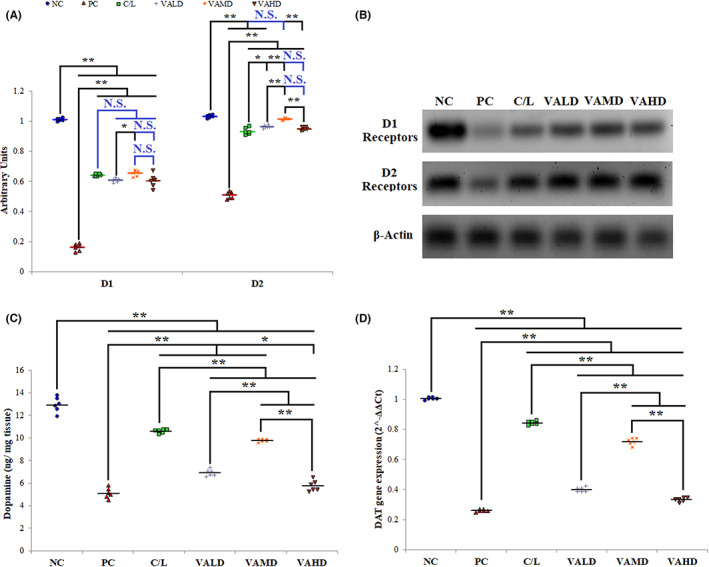
Comparison between the different studied groups according to their efficacy in generating dopaminergic neurons. (A) Comparison between different groups according to quantitative analysis of Western blot bands intensities of brain dopaminergic receptors expression, (B) Western blots for brain dopaminergic receptors, (C) Effect of different treatment groups on dopamine levels in the brain (ng/mg tissue), (D) Effect of different treatment groups on DAT gene expression assessed by qRT‐PCR. ANOVA test was used to compare between the different groups with post hoc test (Tukey) and post hoc test to compare different receptors for the same group. *: Statistically significant at *p* ≤ 0.05, **: Statistically significant at *p* ≤ 0.001, NS: Statistically non‐significant (*p* > 0.05), *n* = 6; all results are presented as mean ± SD. Positive control group received haloperidol 1.5 mg/kg IP once daily for 35 days and 1 mL corn oil orally starting from Day 21. All drugs or vehicles were given as daily 1 mL oral injection for a treatment period of 14 days starting from Day 21 of induction. VALD, VAMD, and VAHD were given at doses of 1500, 3000, and 4500 IU/kg/day vitamin A orally dissolved in corn oil. C/L, carbidopa/levodopa; DAT, dopamine transporter; NC, normal control; PC, positive control; VAHD, vitamin A‐high dose; VALD, vitamin A‐low dose; VAMD, vitamin A‐medium dose. Original blots are provided in the supplementary figures; SF1–SF8.

Induction of PD significantly decreased brain levels of D1, D2, DA, and DAT when compared to the normal rats group (*p* ≤ 0.001). The percentage decrease of D1 and D2 expression in brain tissues after induction were 84.2% and 50.5%, respectively. All treatments significantly increased the levels of the four markers of dopaminergic neuron regeneration. The greatest D1 expression among treated groups was observed in C/L‐ and VAMD‐treated groups with no statistically significant difference between them (*p* = 0.989). On the other hand, the VAMD‐treated group showed the greatest D2 expression which was significantly higher than any other treated group (*p* ≤ 0.001), Figure 7. The C/L‐treated group revealed the highest levels of DA and DAT followed by VAMD; both groups showed statistically significant differences compared to VALD and VAHD (*p* ≤ 0.001), Figure 7.

### 3.6 Regeneration confirmation via histopathological examination of substantia nigra

**FIGURE 8:**
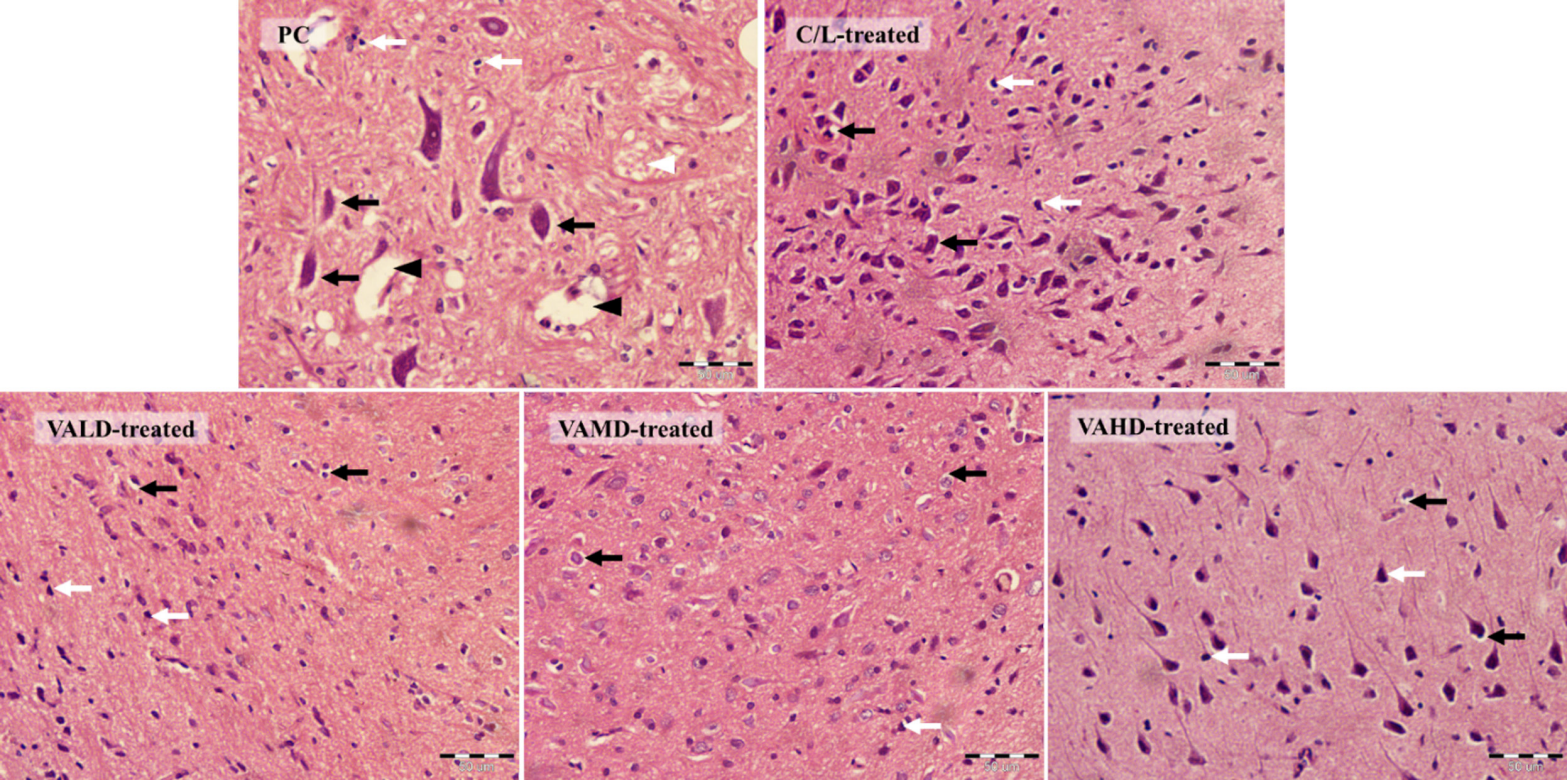
Representative light microphotographs of H&E‐stained sections from the substantia nigra of PC and treated rats ×400. Black arrows point to distorted and degenerated neurons, white arrows point to darkly stained pyknotic neurons, black arrow head points to perivascular edema and white arrow head points to hypoxic‐degenerated cytoplasm. C/L, carbidopa/levodopa; PC, positive control; VAHD, vitamin A‐high dose; VALD, vitamin A‐low dose; VAMD, vitamin A‐medium dose.

Brain sections showing substantia nigra of rats with haloperidol‐induced PD demonstrated a disorganized morphological structure with numerous degenerated, dark‐colored, and shrunken neurons with pyknotic (condensed) nuclei. Sections also demonstrated hypoxic degenerated cytoplasm and perivascular edema. The percentage of degenerated neurons in these sections was 86 ± 10.5% which represents a degeneration score of 4. Haloperidol‐induced neuronal damage was attenuated with treatment with C/L or either dose of VA showing less degenerated neurons. The mean percentages of degeneration in brain sections of rats treated with C/L, VALD, VAMD, and VAHD were 22.6 ± 9.7% (score 1–2), 47.4 ± 7.4% (score 2–3), 18.3 ± 4.5% (score 1) and 35.2 ± 8.7% (score 2), Figure 8.

### 3.7 Influence on PD serum markers and associated motor dysfunction

**FIGURE 9:**
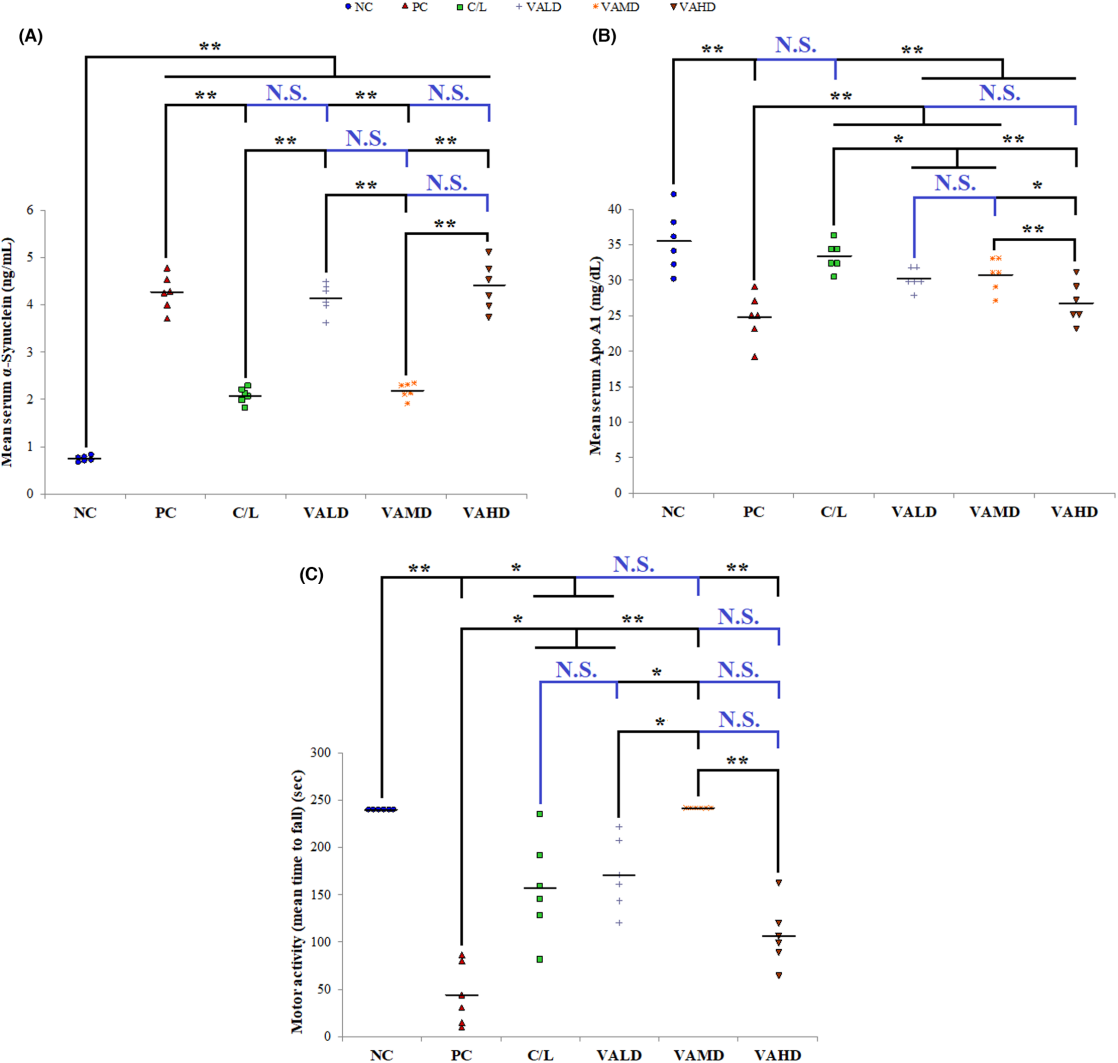
Effect of different studied groups on PD serum markers and motor activity. (A) Effect of different treatment groups on serum α‐Synuclein (ng/mL), (B) Effect of different treatment groups on serum Apo A1 (mg/dL), (C) Effect of different treatment groups on motor activity (mean time to fall in Rota rod assay (sec)). ANOVA test was used to compare between the different groups with post hoc test (Tukey) and post hoc test to compare different receptors for the same group. *: Statistically significant at *p* ≤ 0.05, **: Statistically significant at *p* ≤ 0.001, NS: Statistically non‐significant (*p* > 0.05), *n* = 6; all results are presented as mean ± SD. Positive control group received haloperidol 1.5 mg/kg IP once daily for 35 days and 1 mL corn oil orally starting from Day 21. All drugs or vehicles were given as daily 1 mL oral injection for a treatment period of 14 days starting from Day 21 of induction. VALD, VAMD, and VAHD were given at doses of 1500, 3000, and 4500 IU/kg/day vitamin A orally dissolved in corn oil. Apo A1, Apolipoprotein A1; C/L, carbidopa/levodopa; NC, normal control; PC, positive control; VAHD, vitamin A‐high dose; VALD, vitamin A‐low dose; VAMD, vitamin A‐medium dose.

In the present work, the PC group showed significantly higher serum α‐synuclein levels (ng/mL) and less ApoA1 levels (mg/dL) compared to the NC group (*p* ≤ 0.001). Only C/L and VAMD therapies significantly decreased mean serum α‐synuclein levels and increased serum ApoA1 levels compared to the untreated rats with induced PD (PC group) (*p* ≤ 0.001). Upon comparing C/L‐ and VAMD‐treated groups, there was no statistically significant difference between them in reducing serum α‐synuclein levels (*p* = 0.985), whereas C/L‐treated group showed significantly higher serum ApoA1 levels (*p* ≤ 0.05), Figure 9A,B.

Regarding the influence of different tested therapeutics on motor coordination using the Rota rod experiment, the mean latency to fall (sec) in the untreated diseased rats was significantly shorter than that of normal rats (*p* ≤ 0.001). It was noted that almost all normal rats did not fall during the experimental timing. Different tested therapeutics, except for VAHD, significantly increased latency to fall compared to the PC group. *p* Values for comparing mean time to fall from the rod of C/L‐, VALD‐, VAMD‐, and VAHD‐treated groups and PC were 0.046, 0.026, ≤0.001, and 0.252, respectively. Remarkably, VAMD‐treated rats showed comparable results to normal rats with a non‐statistically significant difference between them in motor coordination (*p* = 1.000), Figure 9C.

## 4 DISCUSSION

PD is a progressive neurodegenerative disease characterized mainly by neuronal degeneration of dopaminergic neurons in the substantia nigra. Though hypodopaminergic status is a key feature of PD, much is still uncovered regarding PD pathology. Involvement of pathological features other than those related to dopamine is supported by the fact that the standard of care; C/L, which acts mainly on dopaminergic pathways, shows only initial effectiveness while long‐term use is associated with loss of effectiveness and incidence of side effects.

One of the possible pathogenic causes that were not hypothesized before is that PD is associated with retinoid depletion within the brain tissue. This hypothesis is based on the presence of diffuse stellate cells (V_A_‐storing cells) system and that stellate cells exist mainly in the liver as well as in extrahepatic sites including the kidney, colon, pancreas, lung, and heart.^13^, ^14^ As a response to tissue injury, these stellate cells become activated myofibroblasts that lose their V_A_ storage capacity, and this is the fundamental explanation for hepatic fibrosis pathogenesis.^14^ Within the brain, astrocytes mimic HSCs both structurally and functionally. Further, in response to brain injury, astrocytes become activated in many brain pathological conditions including PD, just like HSCs.^15^, ^16^ Thus, if PD is associated with V_A_ deficiency within the brain tissue, V_A_ supplementation would selectively target the V_A_‐deficient brain.

Herein, PD was pharmacologically induced in rats using haloperidol which induces muscle rigidity that mimics PD‐like symptoms in experimental animals. It is simpler as compared to the classical 6‐hydroxydopamine model. Haloperidol blocks striatal dopamine transmission resulting in abnormal downstream firing within the basal ganglia circuits that is evident as symptoms of muscle rigidity and catalepsy. In addition, haloperidol reduces the striatal content of DA, noradrenaline and 5‐HT. However, acute haloperidol administration causes only PD‐like movement disorders without causing nigro‐striatal degeneration observed with neurotoxic models.^29^ Brain injury can be observed histologically when haloperidol is chronically administered for durations starting from 7 days,^30^ and repeated administration of haloperidol for 21 days results in a dramatic decrease in the number of physiologically active DA neurons in the substantia nigra indicating massive degeneration.^31^ In the current investigation, haloperidol was administered for 35 days, based on data obtained from a pre‐conducted pilot study as this duration caused persistent motor incoordination and displayed significant degeneration in the substantia nigra.

In order to prove our postulated hypothesis, rats with induced PD were supplemented with multiple oral doses of fluorescently labeled V_A_‐coupled liposomes (FVL). Tracing the bio‐distribution of these liposomes in multiple harvested organs by quantification of Nile red fluorescence would indicate the amount of V_A_ uptaken by various organs. The organ with the greatest fluorescence intensity was the brain which far exceeded the detected fluorescence in any other organ. Other organs with detectable fluorescence were the small intestine then the liver. These observations may indicate that brain injury during PD is associated with V_A_ deficiency. Some preclinical studies are in support of our results; for example, V_A_‐deficient rats have motor impairments analogous to those observed in rats with induced PD or in mice lacking retinoid receptors.^32^ Moreover, another study reported that V_A_ deficiency diminished dopaminergic transmission.^33^ Since, as proved in our previous work, orally administered V_A_ is primarily stored within intestinal stellate cells (an extrahepatic V_A_ –storage site) that can direct their V_A_ load toward V_A_‐deficient sites (the liver in case of hepatic fibrosis).^19^ This means that if PD pathogenesis is associated with retinoid depletion within the brain, the brain would extract V_A_ from the intestine following oral administration to compensate for V_A_ deficiency. Furthermore, these observations indicate efficient brain tissue targeting by orally administered V_A_ in the case of PD.

Contrariwise, in normal healthy rats, the small intestine showed the maximal fluorescence intensity, followed by liver sections and only minimum fluorescence was detected in any other organ, including the brain. This indicates that FVL were considerably stored in the intestinal stellate cells, the main V_A_‐storage organ in normal rats with orally fed V_A_.^34^ Consistent with these results, whole animal photon imaging revealed entrapment of orally administered V_A_‐coupled liposomes mostly in the small intestine of normal rats for 6 days and minimally detected in other organs including the liver.^19^ Green and Lewis et al. have previously explained the link between V_A_ storage sites in V_A_ sufficiency/deficiency states.^35^, ^36^

To verify that FVLs were selectively accumulated within reactive astrocytes (that resemble activated HSCs) in brain tissue sections of rats with induced AD, activated astrocytes were immunofluorescently labeled using a‐SMA antibody as a selective marker for these cells.^37^ Confocal laser microscopy micrographs of brain sections revealed a coincidence of FVLs and stained activated astrocytes, indicating that these cells may act as reservoirs for V_A_ within the brain and loss of their V_A_‐storage capacity in PD status. Consequently, orally fed FVL preferentially accumulated within these V_A_‐deficient cells. In support of this conclusion, it has been proposed that astrocytes could be the source of RA for neurogenesis regulation in the adult brain.^17^, ^18^ Activated astrocytes were found diffusely distributed within the brain tissue. In accordance with this observation, Sofroniew and Vinters reported that astrocytes tile the entire CNS and that there are no CNS regions devoid of this type of cells.^38^ Taken together, V_A_ can target activated astrocytes in PD, likewise, it has long been used for targeting activated HSCs in liver fibrosis.

For further confirming the incidence of brain V_A_ deficiency during active PD and correlating brain V_A_ content to hepatic content, gene expression of brain and liver RBP was assessed. In the present work, under normal conditions, hepatic RBP expression was higher than the brain as the liver is the main V_A_‐storage site. Administration of haloperidol for the induction period significantly reduced both brain and hepatic RBP gene expression with more pronounced hepatic RBP reduction. The decrease in hepatic RBP supports the hypothesis of the existence of homeostatic mechanisms working between V_A_‐storage pools and that V_A_‐rich storage sites supplement V_A_‐deficient sites with V_A_ till reaching equilibrium.^19^ In the current scenario, haloperidol induces a state of V_A_ deficiency in the brain tissue that triggered V_A_ extraction from V_A_‐rich sites (the liver) causing hepatic V_A_ deficiency that was not compensated for from any source. Since RBP gene expression levels were assessed during active disease status, this did not provide sufficient time for equilibrium to occur and the percentage reduction of hepatic RBP exceeded that in the brain. In support of our results, Vairetti et al.^39^ argued the presence of cross‐talk between CNS and liver and proved impaired hepatic function in rats with 6‐hydroxydopamine‐induced PD. Authors related this cross‐talk to thyroid hormones. Unexpectedly, C/L therapy caused a slight increase in both hepatic and brain RBP gene expression. This may indicate that brain tissue healing by C/L and deactivation of the reactive astrocytes with partial restoration of their V_A_‐storage capacity. In line with this explanation is a study proving that levodopa exerts its neuroprotective effect on dopaminergic neurons via astrocytes.^40^ On the other hand, it was expected that oral supplementation with different V_A_ doses would increase hepatic and brain RBP gene expression. According to the results of the confocal laser microscope experiment for tracing V_A_ biodistribution after oral administration in normal and PD status, it can be inferred that orally administered V_A_ was temporarily stored in intestinal stellate cells before their direction toward V_A_‐deficient sites, brain and liver.

Loss of hepatic retinoid is a characteristic feature of HSCs activation and fibrous deposition within the liver.^41^ Thus, with the purpose of authenticating that PD triggered hepatic V_A_ deficiency with a subsequent incidence of liver fibrosis, fibrous deposition was assessed both biochemically and histopathologically. According to our findings, haloperidol‐induced PD was associated with significant fibrous tissue deposition, representing Ishak score 3, and significantly increased levels of two key fibrosis markers; TGF‐β the strongest inducer of fibrotic diseases and hydroxyproline, a main collagen constituent that acts as a good extracellular matrix accumulation marker.^20^ In accordance, a positive correlation between PD and liver dysfunction was perceived in various clinical studies that confirmed an association between PD and cirrhosis.^42^, ^43^, ^44^ In addition, liver impairment and liver enzymes abnormalities were found to exist in patients with PD.^45^ PD‐associated hepatic fibrosis may be a consequence of brain V_A_ deficiency. In addition to the observed hepatic fibrosis, hypoxic damage was also observed within liver tissue which is, most probably, haloperidol induced as haloperidol was reported to cause liver toxicity.^46^, ^47^ Liver damage induced by haloperidol may augment HCSs activation and exaggerate PD‐induced fibrosis. C/L therapy did not improve the detected hepatic defects caused by PD induction; liver sections did not show neither a significant reduction in fibrous deposition nor fibrosis biomarkers. Clinical reports documented the elevation of serum aminotransferase in up to 9% of patients receiving C/L. Also, some cases of acute liver injury were reported with levodopa.^48^ The toxic effect of C/L is attributed to dopamine metabolism and autoxidation, giving rise to quinones and hydrogen peroxide.^49^ To our knowledge, no study testified the beneficial effects of C/L on liver fibrosis. On the other hand, the effect of orally administered V_A_ on liver fibrosis was dose‐selective; meaning that only the medium dose showed significant efficacy and almost normalized PD‐associated fibrosis. Consistent with our results, oral supplementation of retinyl palmitate, 3000 IU/kg/day (VAMD) retinol oral supplementation to Wister rats with 6‐hydroxydopamine‐induced PD for 28 days exhibited increased retinol content in the liver. Rats pre‐treated with retinol did not present hepatic oxidative damage or thiol redox modifications in liver or altered levels of circulating inflammatory mediators indicating the absence of systemic toxicity to animals.^50^ On the other hand, although VAHD achieved the highest hepatic RBP and was expected to be the most dose capable of reversing HSCs activation and fibrosis, this dose did not reduce fibrosis to any significant level compared to PC group. Most probably, chronic administration of VAHD for the treatment period resulted in hypervitaminosis. Case reports indicated that hypervitaminosis A is associated with hepatocytes injury, necrosis, and HCSs activation with subsequent perisinusoidal and periportal fibrosis,^51^ just like what was observed in our study.

Chronic use (over 5 years) of PD standard of care; C/L is associated with a range of adverse effects including fluctuations, dyskinesias, toxicity, or loss of efficacy.^52^ C/L and all approved PD treatment options are symptomatic rather than curative treatments acting only on dopaminergic pathways despite the involvement of multiple pathways in the pathophysiology of PD.^2^ As a neurodegenerative disease involving degeneration of dopaminergic, as well as motor, serotonergic, adrenergic, and cholinergic neurons,^7^, ^9^ agents with broad‐spectrum regenerative capabilities would be promising curative options. Recent evidence points to the high capacity of many exogenously administered transcription factors (TFs) or miRNAs to reprogram fibroblasts/astrocytes directly to neurons. Among these TFs and miRNAs are ASCL1, LMX1, BRN2, NURR1 FoxA2, and MiR‐124 which were successfully capable to reprogram fibroblasts/astrocytes into different neurons subtypes including dopaminergic, motor, serotonergic, adrenergic, and cholinergic neurons.^7^, ^9^ Many observations highlighted the potential of V_A_ as a regenerative medicine, especially neurodegeneration, since it can promote pluripotent stem cell derivation,^10^ and it is an important neural‐related genes modulator.^12^ It may have an influence on fibroblast reprogramming as an important survival factor for fibroblasts.^11^ Thus, the prospective ability of V_A_ to induce endogenous TFs that are known to reprogram fibroblasts for neuronal regeneration and cure PD was evaluated.

All studies that applied TFs and miRNA for fibroblasts/astrocytes reprogramming into functional neurons used a combination of multiple TFs with or without miRNA that complement and augment the action of each other. In the following few lines is the description of the functional role of each of the examined TFs and miRNA in reprogramming and neuronal regeneration; ASCL1 is the most widely used TF for fibroblast reprogramming into neurons purpose. Previous trials revealed the successful generation of dopaminergic (by combining with NURR1), noradrenergic, GABAergic, and glutamate‐energy neurons, motor and serotonergic with the well‐definite role of this TF in the conversion process.^53^, ^54^, ^55^, ^56^, ^57^ NURR1 was proved to be a key factor for a dopaminergic and noradrenergic neuronal generation; it was evidenced to be the vital determinant of the specification, survival, and maturation of dopaminergic neurons in development and adulthood.^53^, ^58^ Moreover, it promoted the expression of mCherry and significantly increased the released noradrenaline.^57^ LMX1 increased the efficiency of fibroblasts reprogramming into dopaminergic neurons by cooperating with ASCL1 and NURR1(acted as a promoter).^53^ Furthermore, LMX1 was found to be vital for the maturation of serotonergic neurons in the midbrain dorsal raphe nuclei.^56^ FOX A2 Promote conversion of neurons from human fibroblasts into dopaminergic neurons.^59^ BRN2, in the presence of other TFs, successfully converted fibroblasts to dopaminergic and motor neurons.^54^, ^59^ MiR‐124 is a miRNA that controls many genes involved in neuronal differentiation regulation besides its ability to promote neuronal development through class IIa histone deacetylase (neuronal differentiation) suppression.^60^ In motor neurons, MiR‐124 was found to trigger chromatin accessibility, DNA methylation, and reconfiguration of mRNA expression to induce reprogramming,^61^ whereas in dopaminergic neurons, Angelopoulou et al., stated that MiR‐124 could improve ASCL1, NURR1 and LMX1 efficiency to generate TH+ neurons; enhance dopaminergic neurons morphology; surge fibroblasts reprogramming into neurons efficiency.^62^

In the present study, the baseline of these TFs and MiR‐124 before PD induction was high indicating efficient neuronal differentiation and maturation. Induction of PD using haloperidol resulted in a severe decline in TFs known to be involved in fibroblasts reprograming fibroblasts into functioning dopaminergic neurons; ASCL1, NURR1, LMX1, FOX A2, BRN2, and MiR‐124, motor neurons; ASCL1, BRN2, and MiR‐124, serotonergic neurons; ASCL1 and LMX1 as well as noradrenergic neurons; ASCL1 and NURR1, reflecting significant degeneration of diverse neuronal types. C/L therapy significantly increased the levels of all these TFs, especially those involved in reprogramming fibroblasts into motor neurons; BRN2 and MiR‐124. The regenerative ability of C/L is supported by few studies; Kondaveeti et al. demonstrated that magnetically released levodopa from magnetic hydrogels favored the proliferation and differentiation of neural cells (human neuroblastoma SH‐SY5Y cells).^63^ Yet, Thomas et al. verified that levodopa stimulates the production of nerve growth factor and of growth hormone that act complementary to each other for neuronal survival.^64^ Though the apparent efficiency of C/L therapy to inducing reprogramming into multiple neuronal types, especially motor neurons, clinical evidence pointed to the loss of efficacy over time and the development of dyskinesia.^52^ Among all tested V_A_ doses, the medium dose showed the greatest ability to induce the production of all TFs and MiR‐124. VAMD therapy showed significantly higher levels of all TFs than C/L therapy, except for TFs required for reprogramming of fibroblasts into motor neurons, BRN2 and MiR‐124. In agreement, V_A_ is recognized as a common inducer of cell differentiation and can trigger many types of cells to differentiate into neurons.^65^, ^66^, ^67^ There is a strong association between V_A_ and its receptors and PD pathogenesis through retinoic acid receptors RAR/RXR signal transduction pathways; V_A_‐mediated gene transcription regulation is generally induced by RA binding to RAR. RAR belongs to the steroid hormone nuclear receptors superfamily. Upon ligand activation, RAR heterodimerizes with RXR which integrates a unique transcriptional network dependent on retinoid metabolism and controls the function of other nuclear receptors. RXR can also form heterodimers with many key transcriptional sensors including peroxisome proliferator‐activated receptors, the vitamin D receptor, the liver X receptor, farnesoid X receptor, that help maintain homeostasis in many pathways.^68^ Furthermore, former studies clearly indicated that V_A_ deprivation is directly involved in motor neurons pathology.^69^ Other evidence for the strong association between RXR and PD, further ascertaining the potential role of V_A_ in curing PD, includes the ability of RXR to dimerize with NURR1, an orphan nuclear receptor expressed in dopaminergic neurons, so as to co‐regulate NURR1 target genes required for survival and maturation of dopaminergic neurons.^70^ Furthermore, RA controls ALDH1A1 which is an enzyme expressed by dopaminergic neurons for the detoxification of these neurons.^71^

The over‐ability of one specific V_A_ dose to induce regeneration over other doses is consistent with the observation of the effect of retinoids on amphibian limb regeneration, where the author noted that V_A_ effect was concentration‐dependent. This was explained by the author as follows: “Each cell is assigned, by appropriate signals, a positional value which specifies its position with respect to fixed boundary points. The pattern of differentiation of these cells then results from the integration of their positional value according to their genetic constitution and their developmental history. The specification of position can be performed, for example, by a simple gradient of morphogen. At a particular concentration of morphogen cells will differentiate into a particular tissue and at a lower concentration of morphogen cells will differentiate into another tissue”.^72^ This means that though VAMD showed the greatest potential among all tested doses to regenerate brain neurons, this dose may not exhibit similar efficacy for regeneration in other organs.

To further assess the regeneration of dopaminergic neurons, the influence on DA receptors and transporter expression as well as DA production was investigated. Haloperidol resulted in a significant reduction in both D1, D2, DAT expression, and DA confirming significant dopaminergic neurons degeneration. Haloperidol is a selective D2 antagonist. Previous studies examining the effect of haloperidol on D1 and D2 expression show contradicting results with relatively general agreement on haloperidol‐induced D1 down‐regulation.^73^ It was concluded from reviewing literature that haloperidol may cause D2 down‐regulation if administered for a relatively short period and increase D2 when administered for longer periods. Also, the effect on D2 receptors was proved to be region‐specific.^73^, ^74^, ^75^ Restoration of neuronal receptors, transporters and neurotransmitters is indicative of their regeneration.^76^ C/L and all VA doses significantly increased D1, D2, DAT expression, and DA level endorsing efficient dopaminergic neurons regeneration. It was noted that the number of folds of increasing D1 expression achieved by all therapies was greater than D2. A previous study showed that D1 is more implicated in neurogenesis than D2 and this may in part explain our observation.^77^ Most probably, as a precursor for DA production, C/L showed significantly higher levels of DAT and DA than V_A_ therapies rather than an indication of dopaminergic neurons regeneration.

Dopaminergic neurons regeneration was also assessed histopathologically and mean percentages of degeneration in substantia nigra brain sections, as the most affected area of progressive dopaminergic neurodegeneration during PD, was calculated and scored. The results supported efficient regeneration by different therapies. The lowest percentages of degeneration were observed in the VAMD‐treated group followed by the C/L‐treated group which matches our former results. Consequently, both VAMD and C/L therapies significantly improved PD biomarkers; α‐synuclein and ApoA1 and motor coordination as indicated by increased latency to fall in the rotarod experiment. A recent study by Davidi et al.^78^ reported that α‐synuclein associates with RA normally and physiologically; it caused α‐synuclein nuclear translocation in SH‐SY5Y cells to induce RAR, RXR and other nuclear receptors‐mediated gene transcription including the transcriptional targets for NURR1.^78^ The same study indicated that RA‐induced α‐synuclein nuclear translocation in cultured neurons increased the expression levels of two PD‐associated genes with possible implications in PD pathology. However, in the later experiment, cells were incubated for additional 24 h in a standard conditioning medium to allow protein translation which may indicate more cellular exposure to RA.^78^ This may potentiate the idea of concentration dependency in mediating different V_A_ effects. In accordance, the highest tested dose of V_A_ in the present study failed to correct the altered assessed PD markers and motor activity possibly due to influencing the expression of PD‐associated genes. C/L therapy, but not VAMD, was able to return serum APO A1 levels to normal values. This could be expected, as ApoA1 was found to be correlated to DAT binding to DA,^79^ and C/L more significantly increased DAT and DA levels than VAMD. On the other hand, VAMD showed greater improvement in motor coordination than C/L therapy with results comparable to normal rats further indicating its greater ability to regenerate different neuronal types that were deteriorated by PD progression. One limitation of the current study is the assessment of motor deficits by only one test. However, proof of VAMD efficacy was confirmed by other assessments performed throughout the study.

## 5 CONCLUSION

During active PD, reactive astrocytes lose their capacity to store V_A_. These V_A_‐deficient astrocytes may extract V_A_ from their hepatic stores triggering hepatic fibrosis. Oral VAMD could target VA‐deficient sites, the brain and liver. In the brain, VAMD could reprogram brain fibroblasts/astrocytes into different neuronal types, including dopaminergic neurons, with significant improvement in PD symptoms more efficiently than other doses and more than C/L therapy. In liver, VAMD was able to regress hepatic fibrosis.

## AUTHOR CONTRIBUTIONS

All authors have contributions in performing the research experiments, interpretation of results, writing and revising the manuscript. All authors have read and agreed to the published version of the manuscript.

## CONFLICT OF INTEREST STATEMENT

The authors report no financial or personal conflict of interest.

## Supporting information


Supplementary Figures

## Data Availability

All data generated or analyzed during this study are included in this published article and its Supporting Information. RNA sequences analyzed during the current study are available in the GenBank repository, https://www.ncbi.nlm.nih.gov/genbank/, accession numbers are included in Table 1.
