# Supplementary material for: Reactive astrocytes targeting with oral vitamin A: Efficient neuronal regeneration for Parkinson's disease treatment and reversal of associated liver fibrosis
Source: CNS Neurosci Ther. 2023 Mar 22;29(8):2111–28. doi: 10.1111/cns.14179 (PMC10352881; doi:10.1111/cns.14179)
Supplement: Supplementary file 1 — Supplementary Figures [file CNS-29-2111-s001.pdf]

Supplementary figures

Full unedited blot for Figure 6 (SF1-SF5)

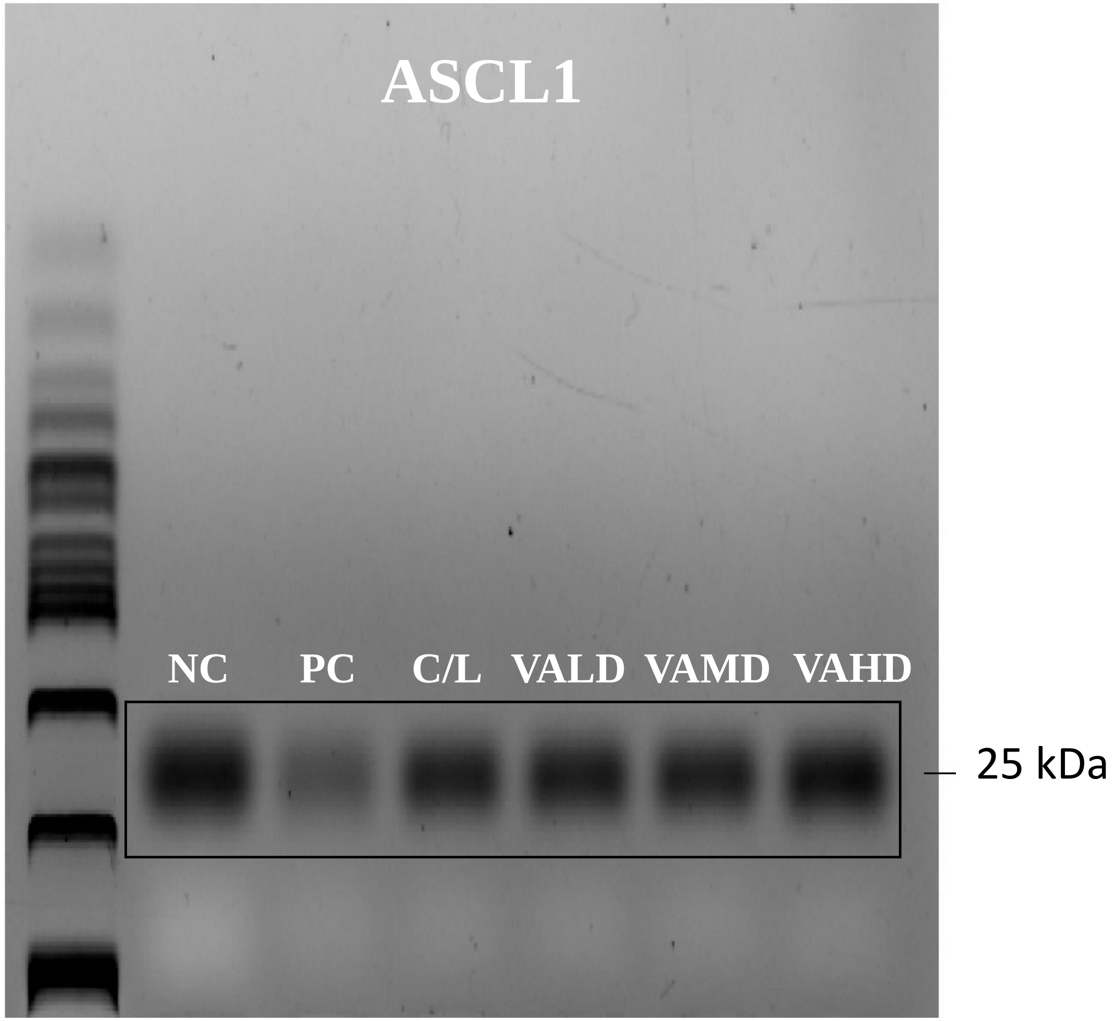

SF1: Western blot for ASCL1. (NC: normal control, PC: positive control, C/L: carbidopa/levodopa, VALD: vitamin A-low dose, VAMD: vitamin A-medium dose, VAHD: vitamin A-high dose)

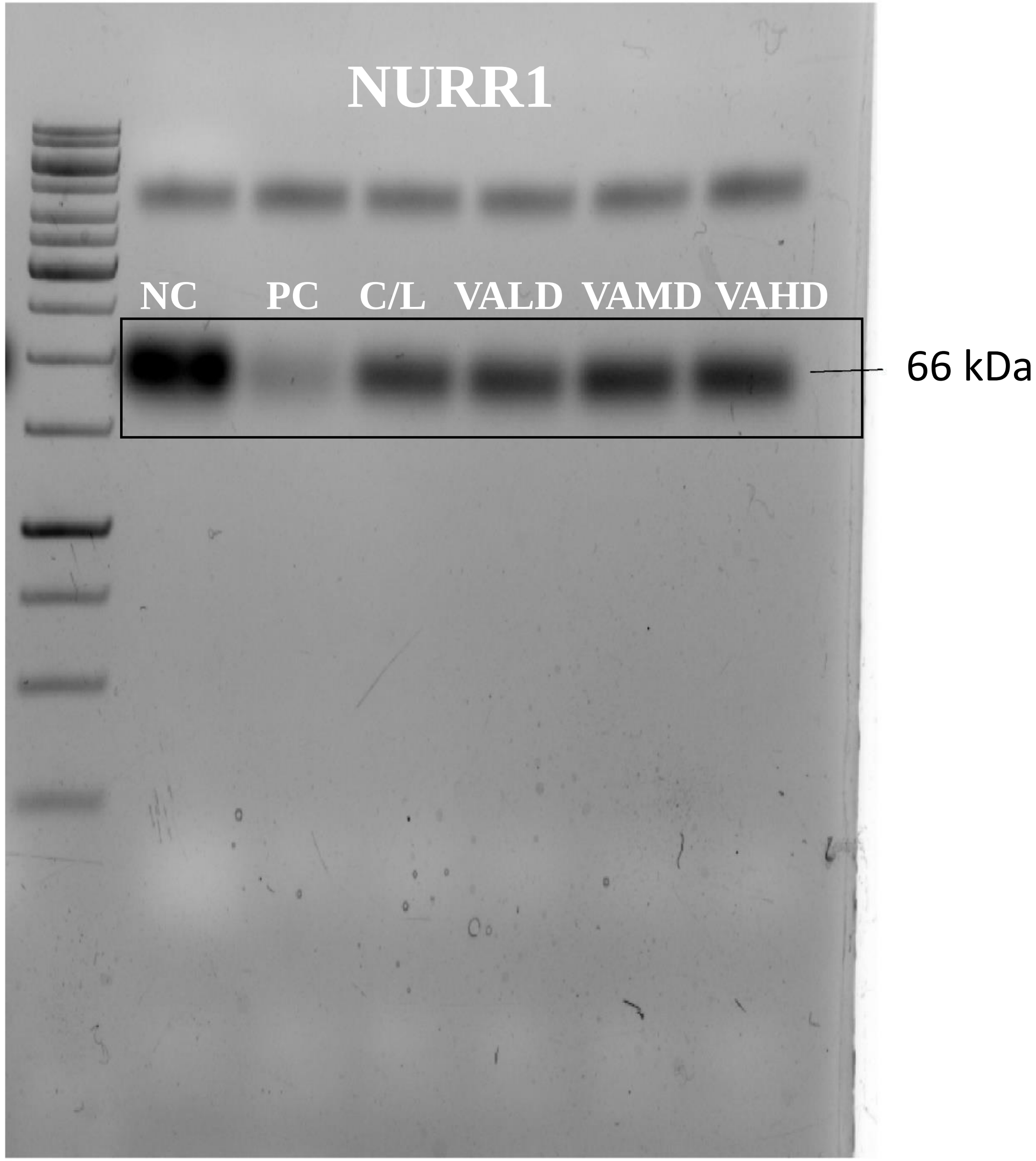

SF2: Western blot for NURR1. (NC: normal control, PC: positive control, C/L: carbidopa/levodopa, VALD: vitamin A-low dose, VAMD: vitamin A-medium dose, VAHD: vitamin A-high dose)

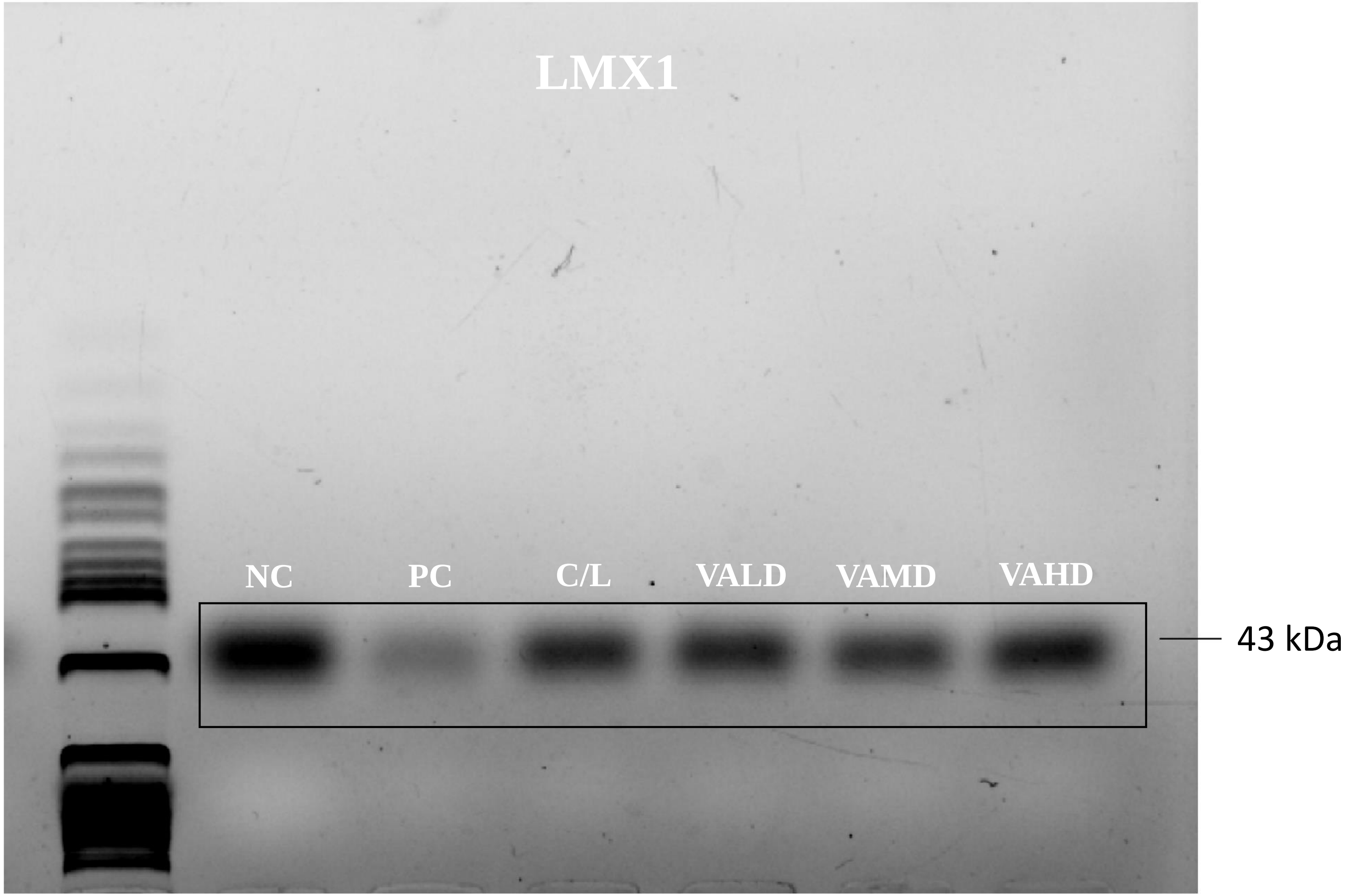

SF3: Western blot for LMX1. (NC: normal control, PC: positive control, C/L: carbidopa/levodopa, VALD: vitamin A-low dose, VAMD: vitamin A-medium dose, VAHD: vitamin A-high dose)

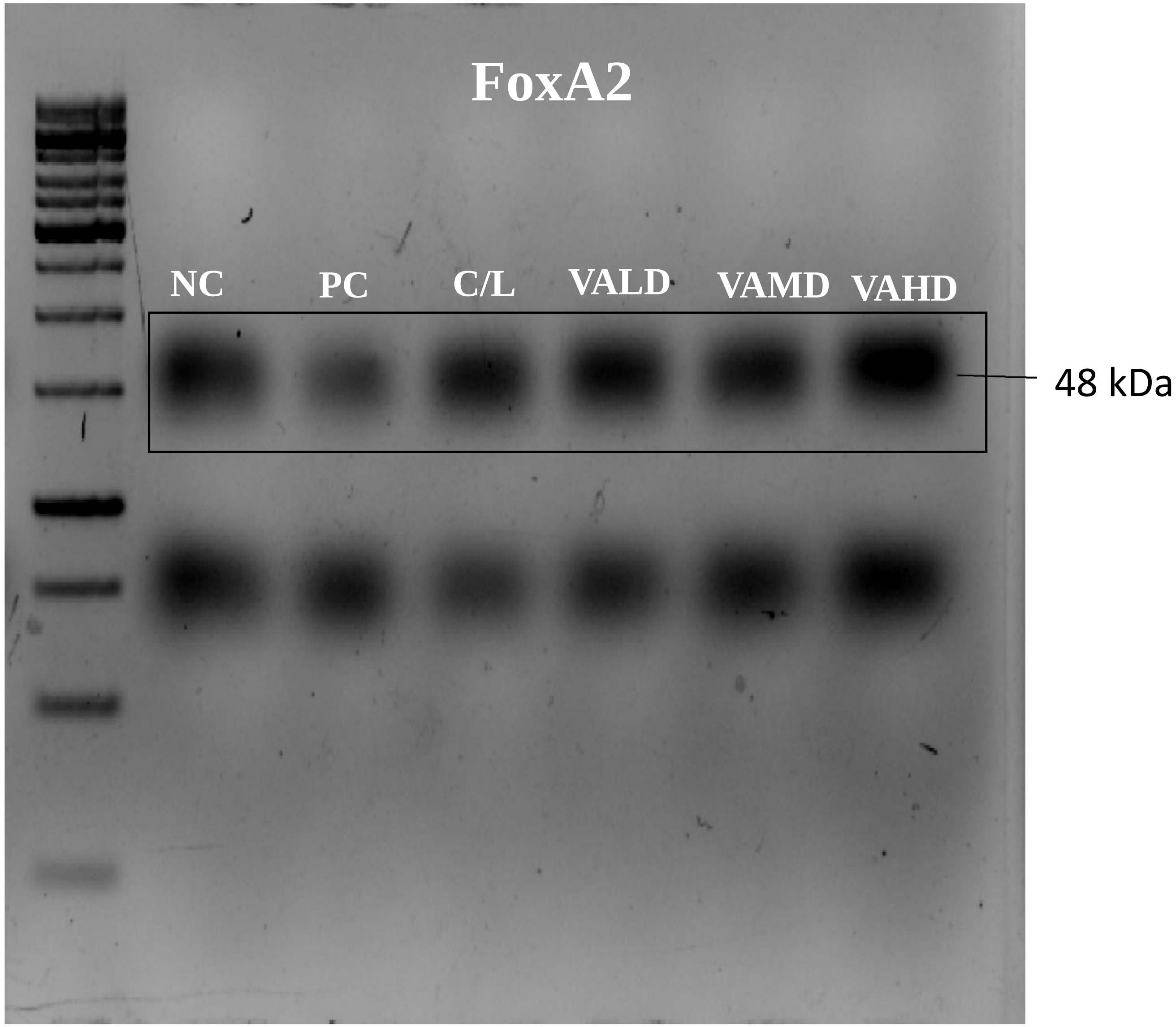

SF4: Western blot for FoxA2. (NC: normal control, PC: positive control, C/L: carbidopa/levodopa, VALD: vitamin A-low dose, VAMD: vitamin A-medium dose, VAHD: vitamin A-high dose)

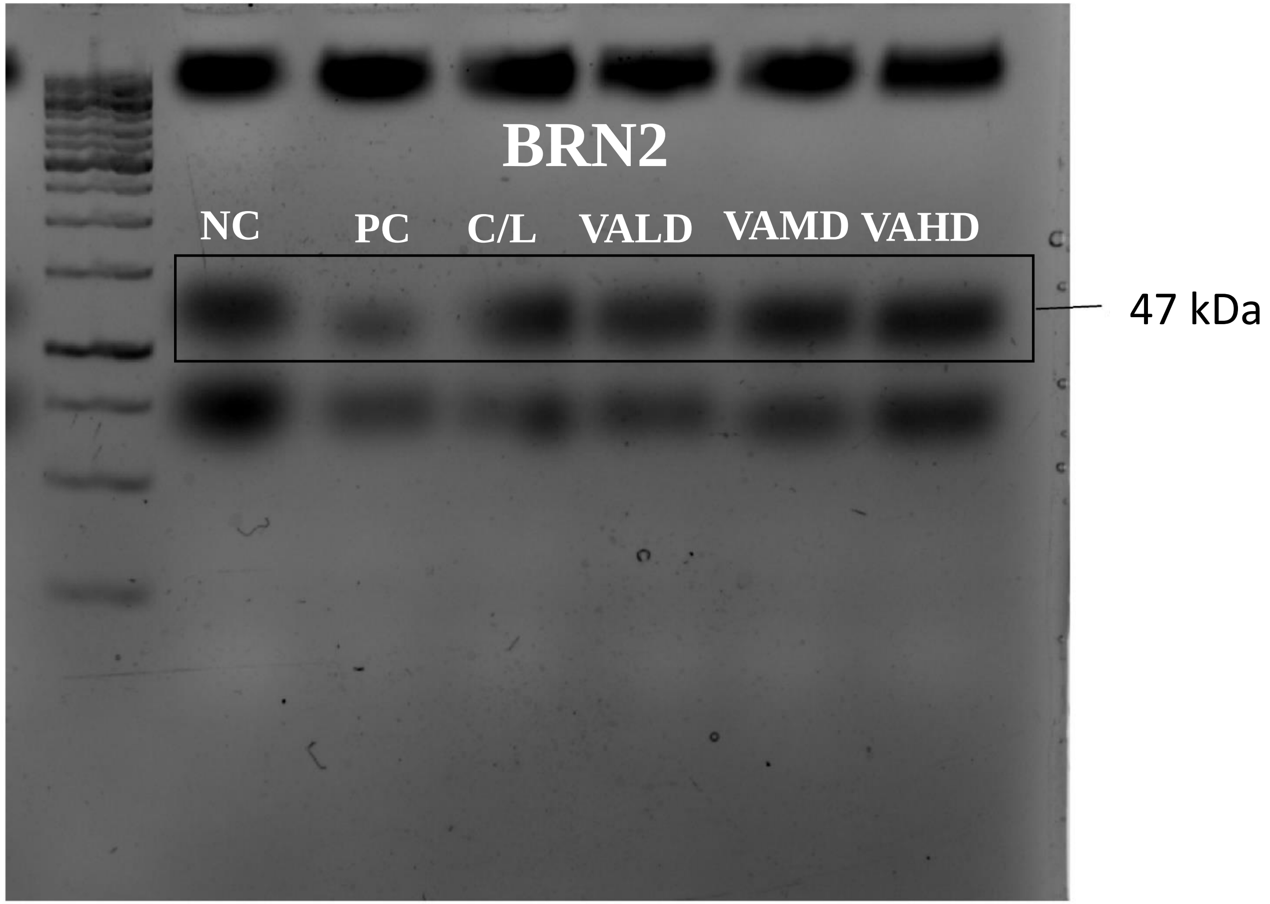

SF5: Western blot for BRN2. (NC: normal control, PC: positive control, C/L: carbidopa/levodopa, VALD: vitamin A-low dose, VAMD: vitamin A-medium dose, VAHD: vitamin A-high dose)

Full unedited blot for Figure 7 (SF6-SF7)

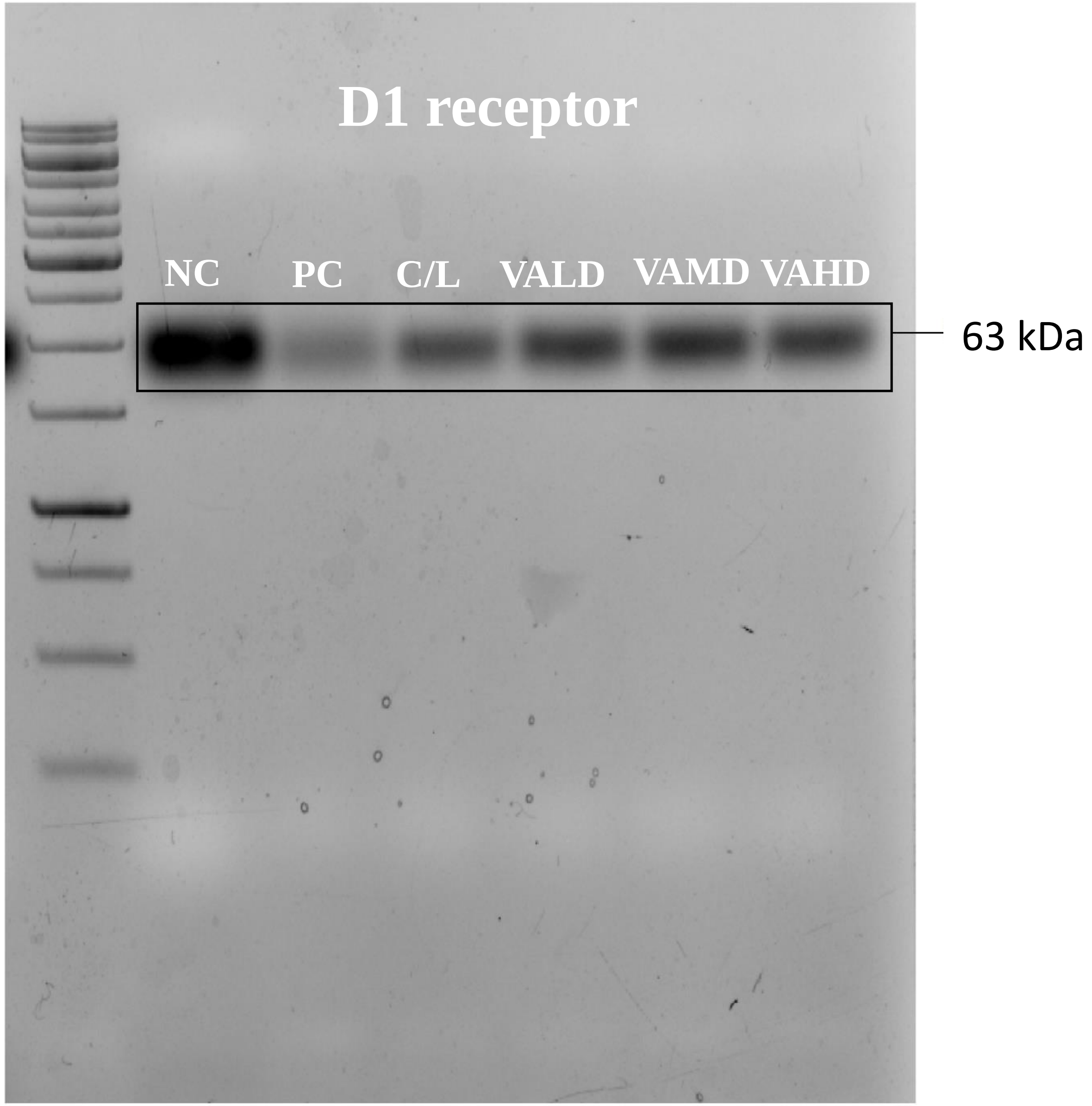

SF6: Western blot for D1 receptor. (NC: normal control, PC: positive control, C/L: carbidopa/levodopa, VALD: vitamin A-low dose, VAMD: vitamin A-medium dose, VAHD: vitamin A-high dose)

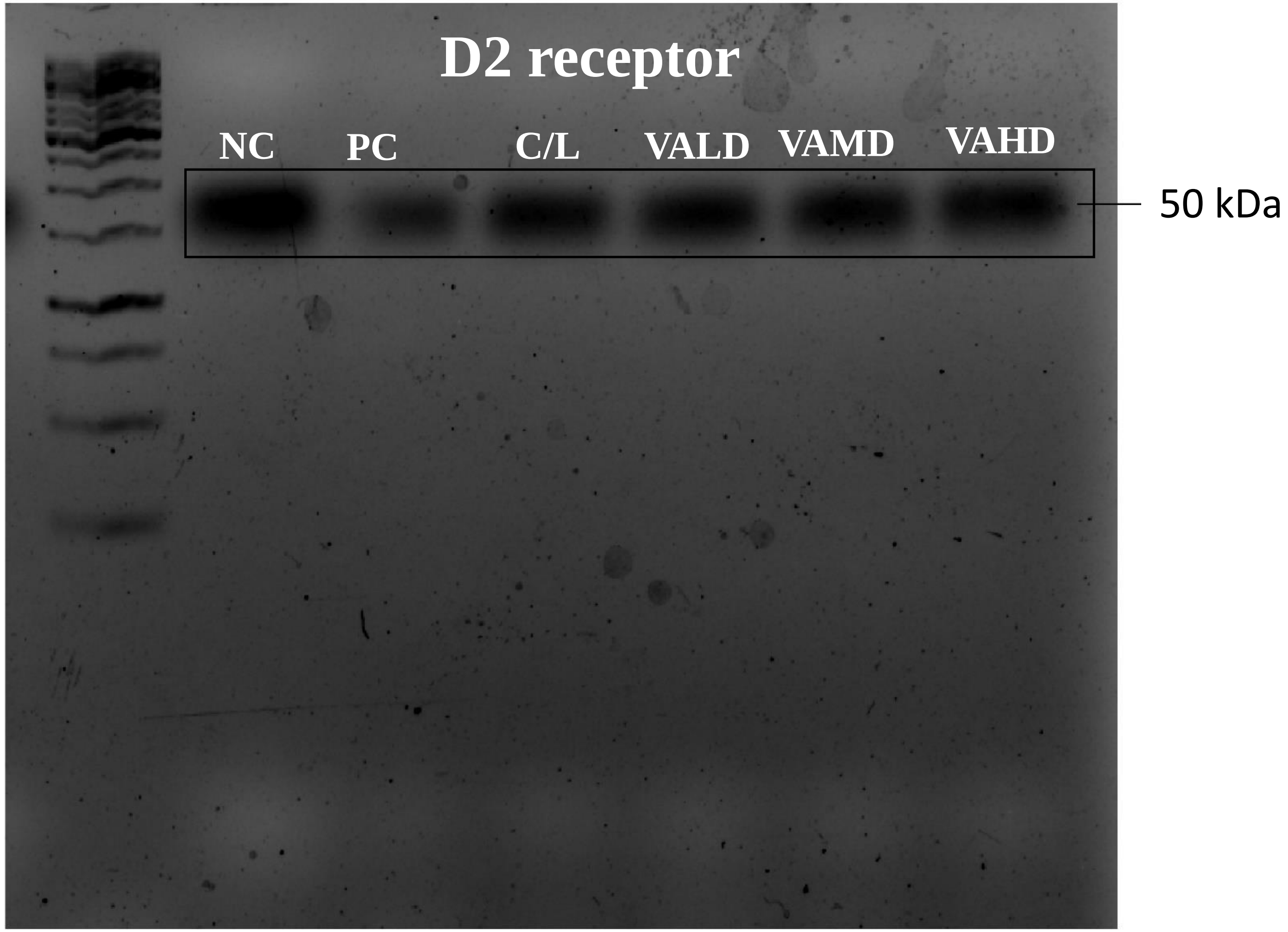

SF7: Western blot for D2 receptor. (NC: normal control, PC: positive control, C/L: carbidopa/levodopa, VALD: vitamin A-low dose, VAMD: vitamin A-medium dose, VAHD: vitamin A-high dose)

Full unedited blot for reference protein (β-actin)

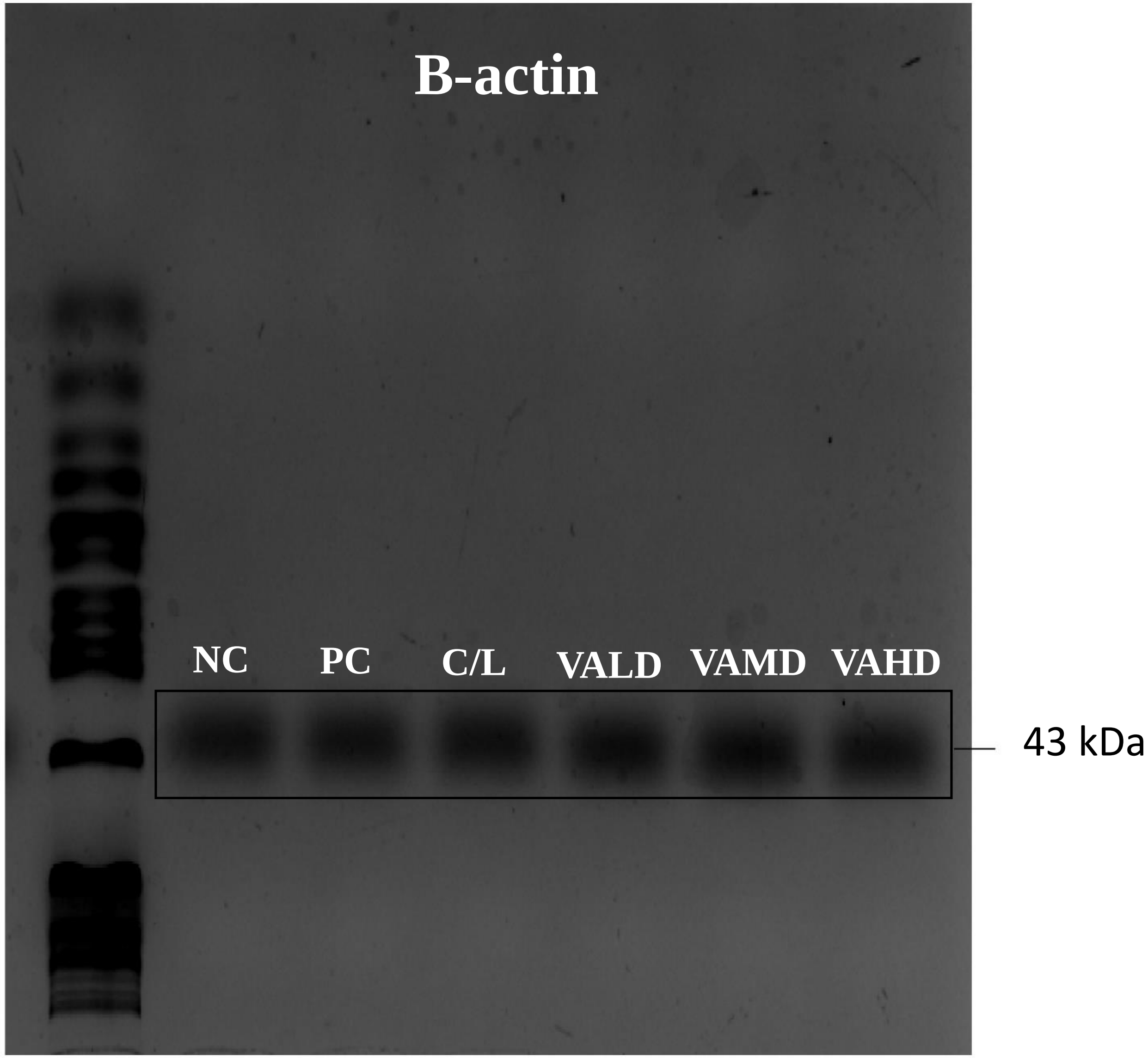

SF8: Western blot for B-actin. (NC: normal control, PC: positive control, C/L: carbidopa/levodopa, VALD: vitamin A-low dose, VAMD: vitamin A-medium dose, VAHD: vitamin A-high dose)
